# Soft Hydroxyapatite Composites Based on Triazine–Trione Systems as Potential Biomedical Engineering Frameworks

**DOI:** 10.1021/acsami.2c16235

**Published:** 2023-01-25

**Authors:** Jinjian Lin, Yanmiao Fan, Daniel J. Hutchinson, Michael Malkoch

**Affiliations:** School of Engineering Sciences in Chemistry, Biotechnology and Health (CBH), Fiber and Polymer Technology, KTH Royal Institute of Technology, Teknikringen 56-58, StockholmSE-100 44, Sweden

**Keywords:** triazine−trione materials, soft hydroxyapatite composites, biomedical engineering, biocompatibility, thiol−ene and thiol−yne materials

## Abstract

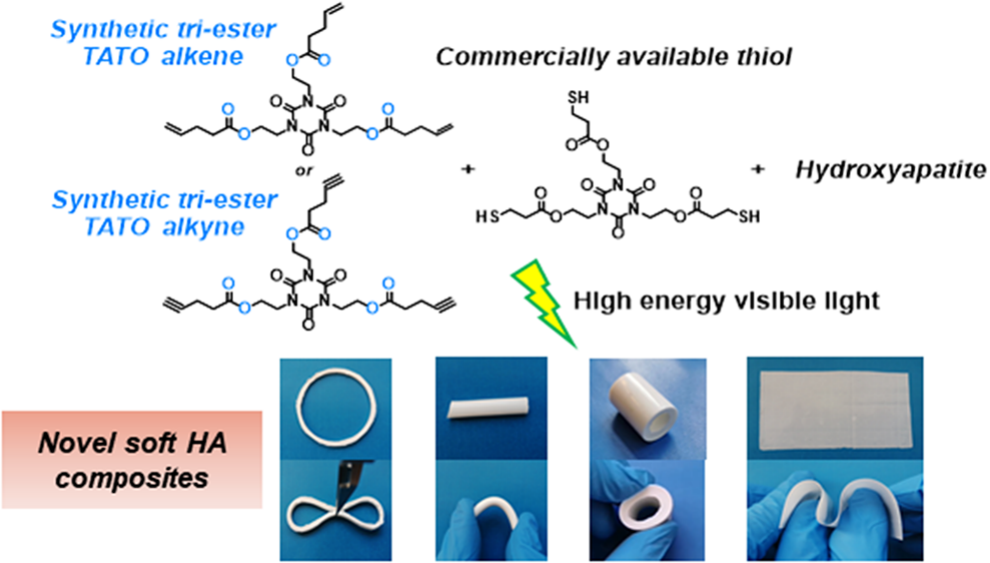

Composites of triazine–trione (TATO) thiol–ene networks and hydroxyapatite (HA) have shown great potential as topological fixation materials for complex bone fractures due to their high flexural modulus, biocompatibility, and insusceptibility to forming soft-tissue adhesions. However, the rigid mechanical properties of these composites make them unsuitable for applications requiring softness. The scope of these materials could therefore be widened by the design of new TATO monomers that would lead to composites with a range of mechanical properties. In this work, four novel TATO-based monomers, decorated with either ester or amide linkages as well as alkene or alkyne end groups, have been proposed and synthesized via fluoride-promoted esterification (FPE) chemistry. The ester-modified monomers were then successfully formulated along with the thiol TATO monomer tris [2-(3-mercaptopropionyloxy)ethyl] isocyanurate (TEMPIC) and HA to give soft composites, following the established photo-initiated thiol–ene coupling (TEC) or thiol–yne coupling (TYC) chemistry methodologies. The most promising composite shows excellent softness, with a flexural modulus of 57 (2) MPa and ε_f_ at maximum σ_f_ of 11.8 (0.3)%, which are 117 and 10 times softer than the previously developed system containing the commercially available tri-allyl TATO monomer (TATATO). Meanwhile, the surgically convenient viscosity of the composite resins and their excellent cytotoxicity profile allow them to be used in the construction of soft objects in a variety of shapes through drop-casting suitable for biomedical applications.

## 1 Introduction

Thermosets and their composites are among the most widely used and versatile engineering materials thanks to the abundant number of different monomers that originate from biomass- or petro-based resources.^1−5^ An example of popular monomers in engineering is the triazine–trione (TATO) family, which includes a wide variety of commercially available building blocks with different reactive groups that result in rigid and stable materials due to the cyclic structure of the TATO ring. This has led to various applications of TATO-based materials in engineering, including thermal insulation materials,^6−8^ magnetic nanoabsorbents,^9^ and coatings.^10,11^ TATO materials have also drawn much attention in the field of biomedical applications. For example, Granskog et al.^12^ presented composites based on tri-allyl and tri-thiol TATO monomers with high concentrations of hydroxyapatite (HA) that could be cross-linked via high-energy visible-light-initiated thiol–ene coupling chemistry (HEV-TEC). These composites were then successfully used to construct shapeable fixation patches for treating bone fractures, which displayed high mechanical performance and exceptional biocompatibility. The use of TEC click chemistry for curing TATO monomers was initially inspired to create suitable alternatives to methacrylate-based dental composites,^13−15^ with the TEC reaction chosen as it displayed the advantages of nonsensitivity to oxygen inhibition, regioselectivity, a high monomer conversion rate, and rapid curing under mild conditions, all of which are beneficial and favorable for curing in a physiological environment.^16−20^

A large variety of TEC thermosets have been described in the literature based on a variety of different monomer architectures. Previous studies include using biomass such as lignin or d-limonene to prepare TEC thermosets as coating applications and also petro-based resources such as ostemer (which is a mixture of allyl, thiol, and epoxy monomers) to fabricate potential devices for organ-on-a-chip applications.^21−24^ Tri-allyl TATO monomer (TATATO) has been used to form thermosets with different ratios of pentaerythritol tetrakis(3-mercaptopropionate) (PETMP), with Young’s moduli ranging from 920 to 1177 MPa.^25^ However, an often-reported technical constraint of the TEC reaction is the limited mechanical properties of materials formed by TEC reactions due to the high flexibility of the thio–ether bonds between monomers^26,27^ This shortcoming can be mitigated through the use of multifunctional alkene and thiol monomers, such as those based on the TATO cyclic architecture. Similar to TEC chemistry, thiol–yne coupling (TYC) belongs to the family of efficient ‘click’ chemistry, and it is another helpful technique in the TATO thiol coupling methodology.^28^ The TYC click reaction results in networks with higher cross-link densities than the TEC reaction due to the alkyne’s ability to react with two thiols instead of one. Consequently, the use of TYC can result in materials with higher strength and rigidity than their TEC counterparts.^29,30^ In another context, TYC materials have been applied to generate interpenetrating soft hydrogel networks or functionalize films or fibers.^31,32^

The stiffness of TEC and TYC materials can also be enhanced through the addition of inorganic filler particles, such as glass particles, HA, and tricalcium phosphate. However, the increase in stiffness is often accompanied by an increase in brittleness which can limit the scope of these composites. In addition, the established materials’ mechanical performance has only covered a small fraction of the vast range of the mechanical behavior of soft or hard human tissues. For example, the HA–TATO composites reported by Granskog et al.^12^ had a flexural modulus of 6.1 GPa. The elastic moduli for soft human tissues including cartilage, ligament, and tendon reach 2–33 MPa,^33^ 25–93 MPa,^34^ and around 1.2 GPa,^35^ respectively, which are much lower than that of the reported HA–TATO composites. Additionally, harder tissues such as bones have a large range of moduli depending on their types. For instance, the elastic modulus of vertebral bone, which is the main component of vertebra, is between 10 and 900 MPa,^36^ while the human proximal tibia bone (trabecular) can display a modulus up to 33.9 GPa.^37,38^

**Figure 1:**
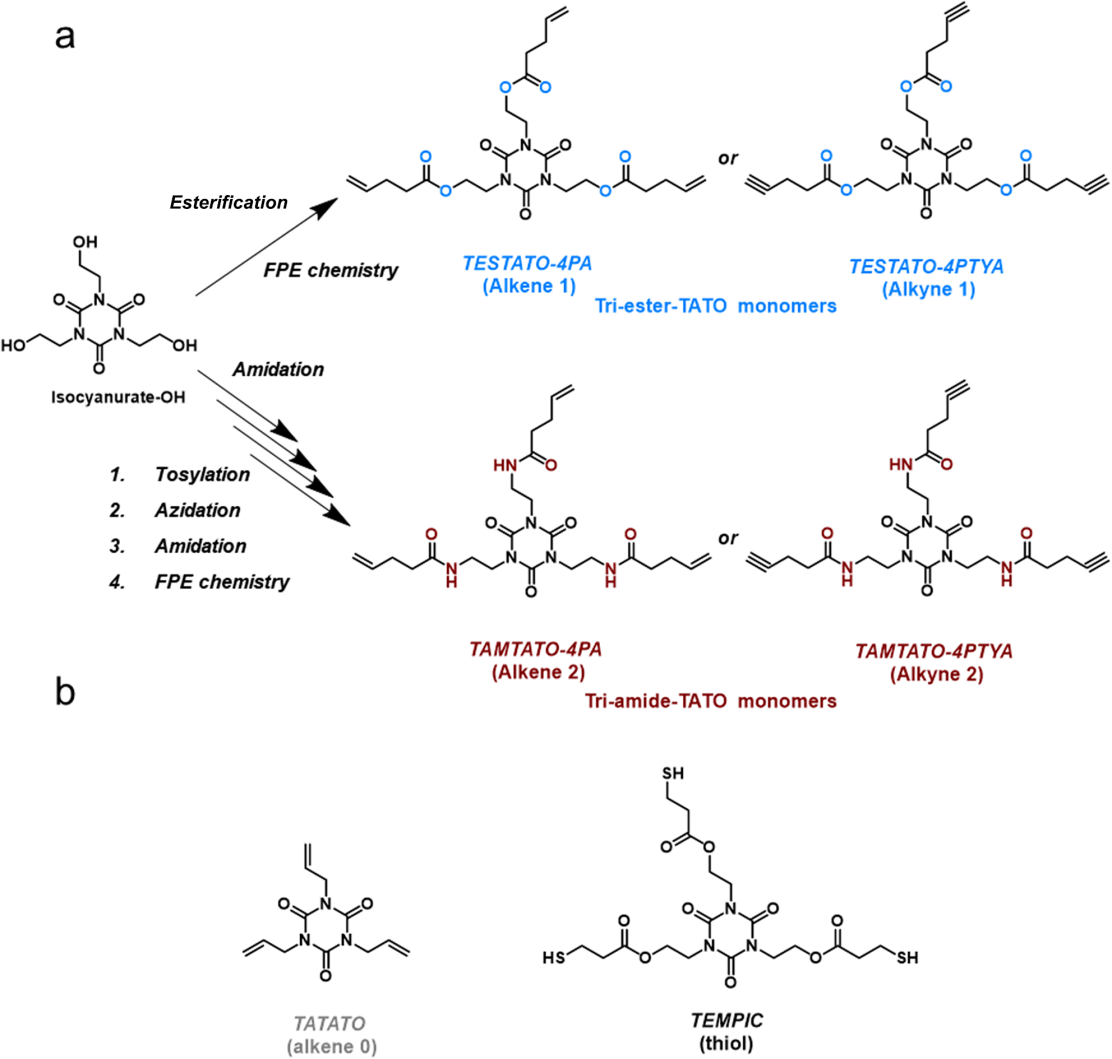
(a) Four novel TATO-based unsaturated monomers with either ester or amide linkages and alkene or alkyne bonds were synthesized through fluoride-promoted esterification (FPE) chemistry. (b) Mechanical properties of the materials made with these novel monomers were compared to those of the commercially available and previously described **alkene 0** monomer. All materials used the commercially available **thiol** monomer.

The large differences between the modulus of these natural tissues and the reported TATO composites show that there is great potential for broadening the scope of TATO composites in biomedicine by increasing the tuneability of the mechanical properties of these composites. We hypothesized that modifying the TATO monomers by introducing additional linkages and space between the unsaturated groups and the TATO ring would yield composites with a wider variety of mechanical properties (Figure 1). Consequently, ester and amide bonds were sought out as potential linkages with the original intent of adding secondary H-bonding to the cross-linked network. Simultaneously, the addition of these groups would elongate the chains between the TATO rings, which was expected to decrease the cross-link density of the network and increase its flexibility; factors that could soften the materials. The use of alkyne groups instead of allyl groups was expected to increase the stiffness of the composites due to the increased cross-link density attained through TYC chemistry. However, these small changes to the TATO monomer structures resulted in significant impacts on the softness of the resulting composites, which we explore here, resulting in materials that display a wider range of mechanical properties than those previously described for TATO systems.

Herein, we report the successful synthesis of novel tri-alkene or tri-alkyne TATO monomers containing either ester or amide linkages by using fluoride-promoted esterification (FPE) chemistry, as well as the first attempts to formulate soft hydroxyapatite–TATO (HA–TATO) composites along with the commercially available and previously reported tri-thiol monomer tris[2-(3-mercapto propionyloxy ethyl] isocyanurate (**thiol**, **TEMPIC**). Compared to the previous reports on thermosets and composites based on the TATO system, extending the distance between the TATO rings and the unsaturated allyl or alkyne groups in tandem with the inclusion of ester and amide bonds was found to greatly impact the formulation and mechanical properties of TATO thermosets and HA-containing composites.

## 2 Experimental Section

### 2.1 Preparation of Materials

The **thiol** monomer TEMPIC was obtained from Bruno Bock Chemische Fabrik GmbH & Co., KG. The other chemicals were obtained or purchased from Sigma-Aldrich Sweden AB, TCI Europe NV and VWR Chemicals. The synthesis procedures and characterization of the tri-ester TATO monomers and tri-amide TATO monomers have been provided in the Supporting Information, Section 1.

### 2.2 Nuclear Magnetic Resonance (NMR)

Analysis was performed using a Bruker 400 ultrashield NMR spectrometer. ^1^H and ^13^C NMR results were collected at a frequency of either 400 or 101 MHz, respectively. For ^1^H NMR, a spectral window of 20 ppm, a relaxation delay of 1 s, and 128 scans with automatic lock and shimming at a concentration of 15 mg/mL have been applied. For ^13^C NMR, a spectral window of 240 ppm, a relaxation delay of 2 s, and 512 scans with automatic lock and shimming at a concentration of 150 mg/mL have been applied. The obtained spectra were analyzed using Mest ReNova version 9.0.0-12821.

### 2.3 MALDI-TOF-MS Analysis

A Bruker UltraFlex L matrix-assisted laser desorption ionization time-of-flight mass spectrometry (MALDI-TOF MS) system with a SCOUT-MTP ion source (Bruker Daltonics, Bremen) was used. All spectra were acquired using a reflector-positive mode. The instrument was calibrated by using SpheriCal TM calibrants obtained from Polymer Factory Sweden AB. Matrixes were prepared by the dissolution of the matrix substance at a concentration of 10 mg/mL, salt at a concentration of 1 mg/mL, and analyte at a concentration of 1 mg/mL in THF. The samples were prepared at a ratio of 20:5:5 of the matrix substance, monomer analyte, and counter ion, respectively. The matrix substance 2,5-dihydroxybenzoic acid (DHB) and the counterion salt sodium trifluoroacetate (NaTFA) were used for mass analysis unless otherwise stated. The received spectra were analyzed with FlexAnalysis Bruker Daltonics, Bremen, version 2.2.

### 2.4 Formulations and Curing of the TEC and TYC Materials

The formulations of TEC and TYC materials were performed by mixing the thiols and different alkene or alkyne monomers along with TPO in optimized ratios (Supporting Information, Section 4) to give thermoset resins. For the preparation of composite resins, the same formulas as the thermoset resins were followed, while hydroxyapatite was added additionally as a filler. The viscous mixtures were thereafter cured in a beam-shaped silicon mold with dimensions of 32 mm × 6 mm × 2 mm by a portable high-performance curing LED lamp (Bluephase 20i, Ivoclar Vivadent AG, Leichtenstein) with a spectrum wavelength of 385–515 nm and light intensity of 2000 mW/cm^2^. LED treatment with 10 pulses (5 s/pulse) was applied on each surface of the **Ene0-T1**, **Ene0-C4**, **Ene1-T1**, and **Ene1-C4** beams, and 15 pulses (5 s/pulse) were used on each surface of the **Yne1-T1** and **Yne1-C4** materials to give full conversion of monomers.

### 2.5 Raman Spectroscopy

The conversions of TEC and TYC monomers were monitored by a portable i-Raman Plus spectrometer (model: BWS465-785S, B&W TEK). The resins were tested with 48 scans (laser wavelength: 785 nm, laser power: 100%, and integration time: 1000 ms), and the cured materials were analyzed with 16 scans (laser wavelength: 785 nm, laser power: 100%, and integration time: 1000 ms). BWSpec software was used to collect the data which was then analyzed using Origin 9.1. The spectra were normalized by the carbonyl shift (1760 cm^–1^). For the TEC materials **Ene0-T1**, **Ene0-C4**, **Ene1-T1**, and **Ene1-C4**, the shifts of the thiol groups (2575 cm^–1^) and C–C double bonds (1645 cm^–1^) were analyzed to confirm the almost full conversion of the reactions. As for the TYC materials **Yne1-T1** and **Yne1-C4**, the shifts of the thiol groups (2575 cm^–1^) and C–C triple bonds (2120 cm^–1^) were analyzed. At least two different batches of resins for each material were tested. The spectra for all the composites and thermosets are given in Supporting Information, Figure S11.

### 2.6 Scanning Electron Microscopy and Energy Dispersive Spectroscopy

The SEM and EDS images of the materials’ cross sections were captured by FE-SEM S-4800 (Hitachi, Japan) and EDS X-Max 80 SDD (Oxford Instruments, UK) systems, respectively. To obtain natural cross sections for characterization, intact composites or thermosets were cryofractured by liquid nitrogen for 5 min, and were then fractured in the middle of the beams. The SEM and EDS analyses of the beams’ cross sections were thereafter conducted by the cooperation of FE-SEM and EDS X-Max systems with 15 kV acceleration voltage. The captured images are provided in Supporting Information, Figure S10.

### 2.7 Water Absorption and Solubility Testing of TEC and TYC Materials

Water absorption and solubility testing were conducted on the materials **Ene0-T1**, **Ene0-C4**, **Ene1-T1, Ene1-C4**, **Yne1-T1**, and **Yne1-C4**. The samples were prepared and cured by a Bluephase 20i LED lamp in a disk-shaped mold with a diameter of 12 mm and thickness of 1.5 mm. For each material, five samples were prepared. The samples were then dried in a 50 °C oven for 3 days until their starting dry weight (*m*_1_) stabilized within 0.1 mg. Afterward, the samples were immersed in PBS solution (pH = 7.4) in a 37 °C oven for 7 days, after which their masses stabilized within 0.1 mg. They were then washed with deionized water, blotted with tissue paper, and their weights were measured to give the wet masses (*m*_2_). Thereafter, the samples were dried in a 50 °C oven until they gave a constant dry mass (*m*_3_). The water absorption (*W*_sp_) and solubility (*W*_sl_) of **Ene0-T1**, **Ene0-C4**, **Ene1-T1, Ene1-C4**, **Yne1-T1**, and **Yne1-C4** materials were then calculated, respectively, by eqs 1 and 2, and the results are shown in Supporting Information, Figure S13:
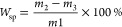
1
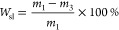
2

### 2.8 Mechanical Evaluations of TEC and TYC Materials

A three-point bending test was conducted on the composites and thermosets under both dry and wet conditions, and the samples were prepared by the method described in Section 2.4. The samples for testing under wet conditions were first immersed in PBS (pH = 7.4) for 7 days at 37 °C. They were then removed from the solution, and tissue paper was used to dry the surface of the materials. The samples were cooled down to room temperature before testing. As for the samples to be tested under dry conditions, their mechanical properties were measured directly after the preparation of the materials. The samples for both dry and wet conditions were then tested with an Instron 5566 universal testing machine (Instron Korea LLC) with a 500 N load cell, a cross-head speed of 1 mm/min, a preload of 0.1 N, and a preload speed of 0.5 mm/min. The center-to-center distance of the lower contacts was set to 30 mm and all measurements were conducted at 20 °C with a relative humidity of 50%. The data were analyzed and collected by Bluehill software. The flexural modulus was calculated by eq 3, where *L* is the lower contacts’ distance, *m* is the slope at the initial elastic region of the load and displacement curve, *w* is the width of the beam, and *d* is the thickness of the beam. For each material, at least five samples were tested.
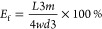
3

### 2.9 Dynamic Mechanical Analysis

A dynamic mechanical analyzer (DMA Q800, TA Instruments, USA) was used to measure the glass-transition temperatures (*T*_g_) and onset points of the composites and thermosets in tensile mode. The materials were of dimensions 12 × 6 × 2 mm (length × width × thickness). The samples were tested under either dry or wet conditions. The samples for testing under wet conditions were first immersed in PBS (pH = 7.4) for 7 days at 37 °C. They were then removed from the solution, and tissue paper was used to dry the surface of the materials. The samples were cooled down to room temperature before testing. A temperature ramp method with a heating rate of 3 °C/min was used, and the testing temperatures ranged from −20, −15, and −10 °C to 80, 90, and 100 °C, depending on the materials’ properties. A strain of 0.1% was induced with a frequency of 1 Hz. To calculate the cross-link density of the thermosets (ρ), the storage modulus (*E*’) at the rubber plateau of the thermosets was assumed as the elastic modulus (*E*) by using eq 4, in which *R* is the Boltzmann gas constant, *T* is the temperature, and the Poisson ratio (*v*) of 0.5 was applied assuming an ideal rubber elasticity. For each material, at least five samples were tested.
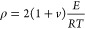
4

### 2.10 Cytotoxicity Assay

Human dermal fibroblast (HDF) and mouse monocyte cells (Raw 264.7) were used for the cytotoxicity assays. Both cell lines were obtained from ATCC and maintained in tissue culture flasks at 37 °C in CO_2_ (5%) with Dulbecco’s modified Eagle’s medium (DMEM), supplemented with fetal bovine serum 10% (v/v), l-glutamine (4 mM), penicillin (100 IU mL^–1^), and streptomycin (100 μg mL^–1^). Cells were harvested and transferred into 96-well plates at a concentration of 1 × 10^4^ cells per well in 100 mL DMEM cultured 24 h before use. To test the cytotoxicity of the polymers, the polymers were dissolved in media at the desired concentrations and were introduced to the cells and incubated for 72 h (37 °C, CO_2_ (5%)). Subsequently, Alamar Blue (10 μL) was added, and incubation was continued for 4 h (37 °C, CO_2_ (5%)). Then, the plate was shaken for 20 s, and finally the fluorescence intensity was measured at ex/em 560/590 nm.

The cytotoxicity of the composites was assessed using human dermal fibroblasts (HDFs) and mouse monocyte (Raw 264.7) cell lines. The cells were harvested and seeded in 96-well plates at a concentration of 1 × 10^4^ cells per well in 100 mL DMEM cultured 24 h before use. The composite was formed with a total surface area of 3 cm^2^ and sterilized under UV light for 20 min. After sterilization, the composite was immersed in 1 mL of cell culure medium (3 cm^2^/mL) and transferred into the incubator for 24 h at 37 °C. 100 μL of the composite elute solution was then added to the 96-well plates and incubated with cells for 72 h. After incubation, Alamar Blue (10 μL) was added, and incubation was continued for 4 h (37 °C, CO_2_ (5%)). The plate was then shaken for 20 s, and the fluorescence intensity was measured at ex/em 560/590 nm. All results are shown as mean ± SD.

### 2.11 Object Demonstrations

Rings, columns, tubes, and thin-film-shaped objects were made directly from the **Ene1-C4** resin and cured by an LED lamp Bluephase 20i in specific silicon molds for different shapes, following the same rationale as in Section 2.4.

## 3 Results and Discussion

To understand the structure-to-property relationship of TATO thermosets and composites impacted by structural changes in the TATO monomers, we sought out the synthesis of novel TATO-based monomers that displayed ester or amide linkages as well as alkene or alkyne cross-linkable groups. FPE chemistry was selected as a robust modification reaction to synthesize this library of TATO monomers. Then, the monomers underwent a screening procedure to formulate resins with the tri-thiol monomer **thiol**.^12^ The resins were thereafter cured by HEV light to give both thermosets and HA-including composites. The resins exhibited similar viscosity to the previously reported formulations based on **alkene 0** and **thiol**, which were optimized for application and surfacing on bone tissue substrates under surgical conditions, that is, thin enough for application via syringe and shaping but thick enough to hold their shape and not flow from the desired tissue site before curing.^12,39^ The materials prepared were then assessed by a series of characterization techniques including three-point bending, DMA, SEM, and EDS as well as cytotoxicity evaluations to investigate the influence of different monomer structures and the impact of adding HA as a filler. Finally, the most promising system was translated to potential customizable biomedical engineering devices to showcase their potential.

### 3.1 Chemical and Mechanical Properties of the Novel TATO Alkene and Alkyne Monomers

Inspired by the commercially available **alkene 0** monomer, four novel multifunctional TATO chemicals (**alkene 1**, **alkene 2**, **alkyne 1**, and **alkyne 2**; Figure 1), which contained either ester or amide linkages and C–C double or triple bonds, have been synthesized successfully by FPE chemistry. The aim of introducing the ester and amide groups was to increase the toughness of the cross-linked network through secondary H-bonding; however, the insertion of these linkages between monomers was expected to also slightly decrease the cross-link density and thereby potentially reduce the stiffness of the materials. To counteract the lowering of cross-linking density and subsequent loss in stiffness, we compared the allyl functional TATO monomers in **alkenes 1** and **2** with their alkyne counterparts **alkynes 1** and **2**, in which the latter would each react with two thiols through TYC chemistry, instead of just one.

**Alkene 1** and **alkyne 1** were synthesized in high purity in a single reaction between 1,3,5-tris(2-hydroxyethyl) isocyanurate (isocyanurate-OH) and either carbonyldiimidazole(CDI) activated 4-pentenoic acid (4PA) or 4-pentynoic acid (4PTYA), respectively. The yields of **alkene 1** and **alkyne 1** were calculated to be 92 and 50%. Both products were dark yellow viscous liquids and were characterized by ^1^H and ^13^C NMR spectroscopy and MALDI (Supporting Information, Sections 1.1 and 1.2). **Alkene 2** and **alkyne 2** were synthesized in a multistep sequence, including tosylation, azidation, and amination, and finally FPE reaction with either 4PA or 4PTYA (Supporting Information, Sections 1.3, 1.4, and 1.5). The tri-amide TATO monomers **alkene 2** and **alkyne 2** were both white powders after purification, and their yields reached 42 and 44%, respectively. The differences in the yield between the tri-ester-TATO and tri-amide-TATO monomers indicated that the reactivity of the hydroxyl groups on the TATO core was higher than that of the amine groups with respect to the CDI-activated acids. The liquid state of the tri-ester-TATO monomers and the solid state of the tri-amide-TATO monomers under room temperature also suggested that the ester linkages in **alkene 1** and **alkyne 1** provided weaker hydrogen bonding than the amide groups in **alkene 2** and **alkyne 2**.

### 3.2 Formulations and Mechanical Properties of TEC and TYC Materials

The commercially available tri-alkene TATO monomer **alkene 0**, novel synthesized tri-ester-alkene TATO monomer **alkene 1**, and tri-ester-alkyne TATO monomer **alkyne 1** were formulated with the commercially available **thiol** to give thermosets and HA composites (Table 1). The formulations were stoichiometrically balanced, with the ratio of unsubstituted carbon bonds to thiols of 1:1 for TEC formulations and 2:1 for TYC formulations. This stoichiometry was chosen to promote the complete conversion of all alkene, alkyne, and thiol groups in the formulation in order to create materials with the highest possible cross-link density. Unfortunately, neat formulations with the amide-containing **alkene 2** and **alkyne 2** monomers were unattainable as they proved too insoluble in the **thiol** at room and elevated temperatures.

**Table 1:** Thermoset and Composite Materials Evaluated in this Work and the Concentration of HA and TPO for Each Material, as Well as the Required Pulses of HEV Light for Full Curing^a^

Materials	Alkene	Alkyne	HA	TPO	Pulses of 5 s HEV	Type
**Ene0-T1**	alkene 0		0 wt %^**a**^	0.25 wt %	5	thermoset
**Ene0-C2**	alkene 0		19 wt %^**b**^	0.25 wt %	5	composite
**Ene0-C3**	alkene 0		37 wt %^**c**^	0.25 wt %	5	composite
**Ene0-C4**	alkene 0		56 wt %^**d**^	0.25 wt %	5	composite
**Ene1-T1**	alkene 1		0 wt %^**a**^	0.25 wt %	5	thermoset
**Ene1-C2**	alkene 1		20 wt %^**b**^	0.25 wt %	5	composite
**Ene1-C3**	alkene 1		40 wt %^**c**^	0.25 wt %	5	composite
**Ene1-C4**	alkene 1		60 wt %^**d**^	0.25 wt %	5	composite
**Yne1-T1**		alkyne 1	0 wt %^**a**^	0.88 wt %	15	thermoset
**Yne1-C2**		alkyne 1	19 wt %^**b**^	0.88 wt %	15	composite
**Yne1-C3**		alkyne 1	39 wt %^**c**^	0.88 wt %	15	composite
**Yne1-C4**		alkyne 1	58 wt %^**d**^	0.88 wt %	15	composite

a **a**, **b**, **c**, and **d** indicate 0, 33, 67, and 100% of the determined maximum HA concentrations for each type of the composites.

Inspired by previously established methods,^12^ thermosets **Ene0-T1**, **Ene1-T1**, and **Yne1-T1** were formulated with **thiol** and either **alkene 0, alkene 1**, or **alkyne 1**, respectively, along with diphenyl (2,4,6-trimethylbenzoyl) phosphine oxide (TPO) as the photoinitiator. HA was then added to each thermoset to create a series of composites. The maximum HA concentration that could be added to **Ene0-T1**, **Ene1-T1**, and **Yne1-T1**, while maintaining a fluid homogeneous mixture, was determined to be 56, 60, and 58 wt %, respectively. At these concentrations of HA, the mixtures had a viscosity that allowed them to be applied and spread on surfaces with a spatula while not being so thin as to run-off the curved surfaces before the curing with HEV light could be completed. This viscosity was therefore ideal for their envisioned use in creating in situ bone fixation patches. Composites of **alkene 0**, **alkene 1**, and **alkyne 1** were also formulated with either 33, 67, or 100% of their maximum HA concentration in order to determine how the concentration of HA affected the mechanics of each material. In total, 12 different formulations were made and investigated that varied due to the presence of (i) ester groups, (ii) alkene or alkyne functionalities, and (iii) HA at various concentrations (Table 1).

**Figure 2:**
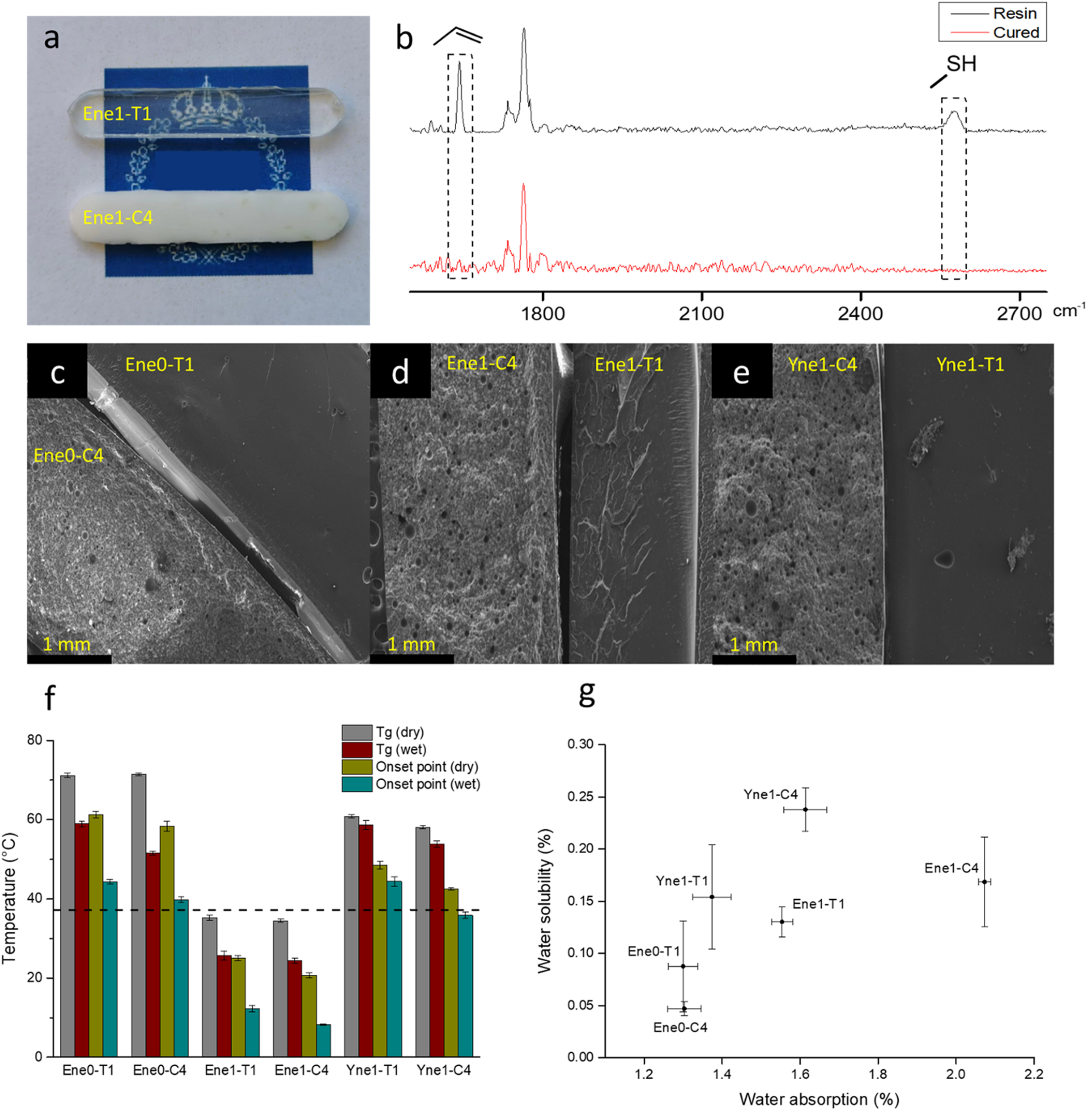
(a) Thermoset and composite beam of **Ene1-T1** and **Ene1-C4**, respectively. (b) Raman spectra of the material **Ene1-C4** before and after curing. (c–e) SEM images of the cross sections of materials **Ene0-T1**, respectively. (f) Dynamic mechanical analysis (DMA) of materials **Ene0-T1**, **Ene0-C4**, **Ene1-T1**, **Ene1-C4**, **Yne1-T1**, and **Yne1-C4** under both dry and wet conditions. Black dashed line indicates the temperature of 37 °C. (g) Water absorption and solubility of materials **Ene0-T1**, **Ene0-C4**, **Ene1-T1**, **Ene1-C4**, **Yne1-T1** and **Yne1-C4**.

After formulation, the mixtures were cured by HEV light from a handheld LED lamp. The TYC **Yne1** materials required a higher concentration of TPO and a longer exposure of HEV light to obtain complete monomer conversion. FT–Raman spectra showed that all the materials had been fully cured as indicated by the disappearance of the thiol signals between 2560 and 2600 cm^–1^ and either the alkene or alkyne signals at 1630–1660 or 2100–2136 cm^–1^, respectively (Figures 2b and S11).

The mechanical properties of these 12 materials were then determined by three-point bending and DMA measurements. The mechanical properties of the three families of materials (**alkene 0**, **alkene 1**, and **alkyne 1**) were found to be significantly different (Table 2). The **alkene 0** family demonstrated the highest flexural modulus and strength, with the **Ene0-T1** thermoset having a modulus of 2723 (30) MPa and a strength of 87 (1). In stark contrast, the modulus and strength of the **Ene1-T1** thermoset were 15 (1) and 1.5 (0.1) MPa, respectively, a remarkable difference considering the only differences between the **alkene 0** and **alkene 1** monomers, were the insertion of an ester linkage and a small increase in distance between the TATO ring and alkene groups. The modulus and strength of the **Yne1-T1** thermoset were 2259 (65) and 71 (1) MPa, which showed that much of the loss in stiffness and strength by introducing the ester linkage was recovered by employing TYC chemistry for cross-linking instead of TEC. The toughness of the **Ene0-T1**, **Ene1-T1**, and **Yne1-T1** thermosets, as calculated from the strain–stress curve, was 2719 (133), 107 (11), and 8192 (322) kJ/m^3^, respectively, while the strain at the maximum stress was 4.4 (0.03), 11.7 (0.4), and 4.6 (0.1)%, respectively.

**Table 2:** Mechanical Properties and Thermal Analysis of the Different **TEMPIC Thiol-Based** Composites and Thermosets under Dry or Wet Conditions^a^

Materials	*E*_f_ (dry) [MPa]	Max σ_f_ (dry) [MPa]	Toughness at maximum σ_f_ (kJ/m^3^)	ε_f_ at maximum σ_f_ (%)	Onset point (dry) [°C]	*T*_g_ (dry) [°C]	*E*_f_ (wet) [MPa]	Max σ_f_ (wet) [MPa]	Onset point (wet) [°C]	*T*_g_ (wet) [°C]
**Ene0-T1**	2723 (30)	87 (1)	2719 (133)	4.4 (0.03)	61 (1)	71 (1)	1599 (66)	37 (3)	44 (1)	59 (0.7)
**Ene0-C4**	6480 (280)	68 (5)	427 (23)	1.2 (0.05)	58 (1)	71 (0.3)	3093 (199)	27 (1)	40 (1)	52 (0.5)
**Ene1-T1**	15 (1)	1.5 (0.1)	107 (11)	11.7 (0.4)	25 (1)	35 (1)	15 (0.4)	1.5 (0.1)	12 (1)	26 (1)
**Ene1-C4**	57 (2)	5 (0.1)	515 (22)	11.8 (0.3)	20 (1)	34 (0.4)	57 (1)	4 (0.1)	8 (0.2)	24 (1)
**Yne1-T1**	2259 (65)	71 (1)	8192 (322)	4.6 (0.1)	48 (1)	61 (0.4)	1663 (66)	43 (1)	44 (1)	59 (1)
**Yne1-C4**	3480 (231)	39 (1)	1942 (98)	3.7 (0.3)	42 (1)	58 (0.4)	2288 (98)	21 (2)	36 (1)	54 (1)

a The values are given as mean, along with the standard error of mean (SEM) in parentheses (*n* > =5).

Despite these large mechanical differences, the cross-link densities of the thermosets, as determined by DMA, were similar (Supporting Information, Figure S12). From **Ene0-T1** to **Ene1-T1**, the cross-link density decreased from 12.3 (0.9) to 10.0 (0.6) *M*_c_, likely due to the elongation of the linkages between monomers from the added ester groups in **Ene1-T1**. As expected, the cross-link density of **Yne1-T1** was higher than that of the TEC thermosets, at 15 (0.2) *M*_c_, but this modest increase of 50% relative to **Ene1-T1** was surprising, as previously reported TATO thermosets have shown a 400% increase in cross-link density when replacing alkenes with alkynes.^30^ It is possible that the higher degree of branching in the **Yne1-T1** cross-linked network, due to each alkyne group reacting with two thiol groups, may explain the large difference in mechanical properties when compared to **Ene1-T1**.

**Figure 3:**
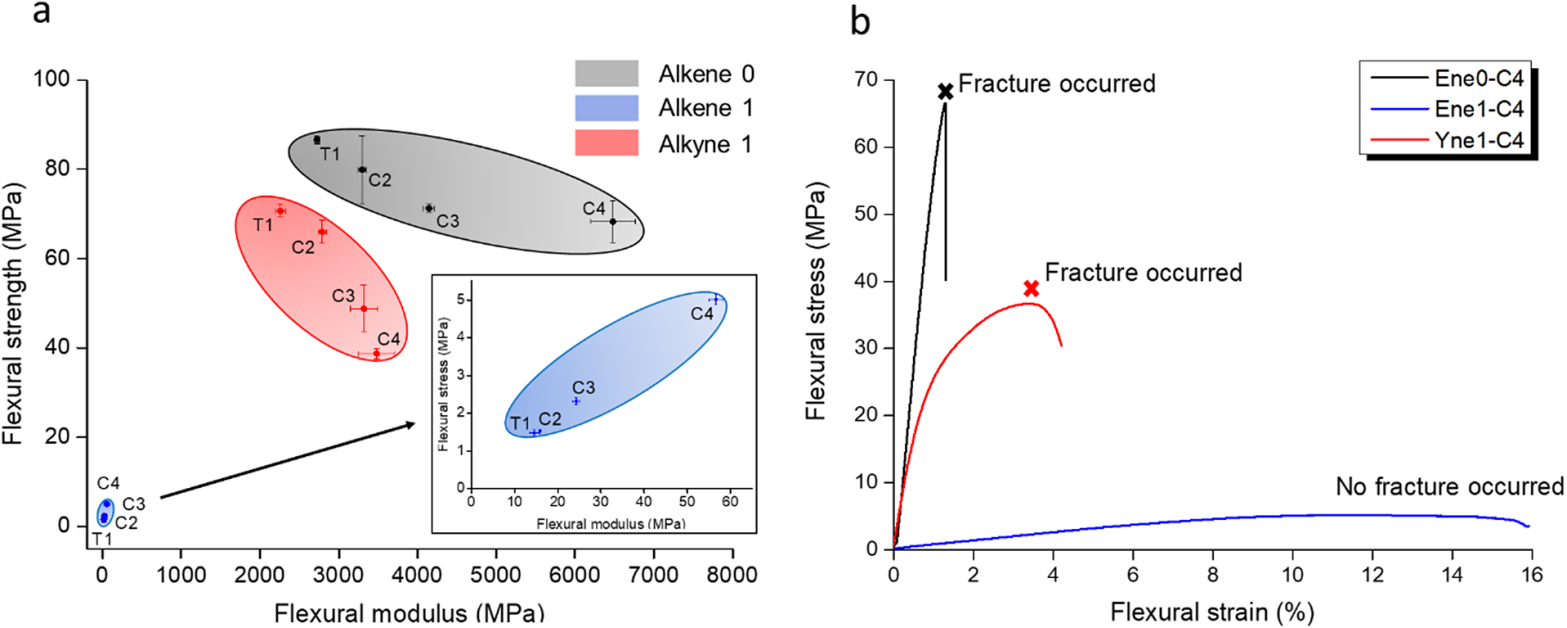
(a) Mechanical property plot with flexural strength vs flexural modulus for 12 different materials evaluated in this study. (b) Flexural strain–stress curves for materials **Ene0-C4**, **Ene1-C4**, and **Yne1-C4**.

The degree to which the mechanical properties of these materials were affected by HA concentration was also very different (Figure 3). Increasing HA concentration had the largest impact on the **alkene 0** materials, with the maximum concentration of HA, 56 wt %, increasing the flexural modulus by 240% to 6480 MPa (**Ene0-C4**). This was accompanied by a decrease in flexural strength to 68 (5) MPa and decreases in toughness, from 2719 (133) to 427 (23) kJ/m^3^, and strain at maximum stress, from 4.4 (0.03) to 1.2 (0.05)%. The **alkyne 1** materials followed this same trend, with the addition of 60 wt % HA increasing the modulus by a more modest 150% to 2259 (65) MPa (**Yne1-C4**), while reducing the strength to 39 (1) MPa. The toughness and strain at the maximum stress of **Yne1-C4** were also lower than those of **Yne1-T1**, but at 1942 (98) kJ/m^3^ and 3.7 (0.3)%, these values were much higher than those of **Ene0-C4**. However, the addition of HA to both the **alkene 0** and **alkyne 1** materials resulted in stiffer but more brittle composites.

The **alkene 1** materials showed a different trend, where the addition of increasing concentrations of HA increased the modulus, while also increasing the strength and toughness and not affecting the strain at maximum stress. For **Ene1-C4**, the modulus and strength were 57 (2) and 5 (0.1) MPa, an increase of 380 and 330%, respectively, while the toughness was 515 (22) kJ/m^3^ and the strain at maximum stress was 11.8 (0.3)%. In this case, stiffness was enhanced by adding HA without introducing brittleness. The reason behind the different responses of the **alkene 1** materials to the increasing concentrations of HA should be investigated further in future studies.

However, the results of composites **Ene0-C4** and **Ene1-C4** suggest that, compared with the previously established composite **Ene0-C4**, the **Ene1-C4** material is statistically more resistant to externally applied energy. Here, it is expected that the values of toughness for the **alkene 1** materials are low due to their much lower flexural modulus and strength, compared with other materials from **alkene 0** to **alkyne 1**. Moreover, when it comes to the discussion about their flexural strain at maximum flexural stress, the **alkene 1** materials showed extraordinary results, which are 11.7 (0.4)% for **Ene1-T1** and 11.8 (0.3)% for **Ene1-C4**. They are 3 and 10 times higher than the ε_f_ values of **alkene 0** materials, which are 4.4 (0.03)% for **Ene0-T1** and 1.2 (0.05)% for **Ene0-C4**, respectively. The alkyne thermoset **Yne1-T1** presented ε_f_ of 4.6 (0.1)%, which is close to the result of **Ene0-T1**. On the other hand, the composite **Yne1-C4** presented ε_f_ of 3.7 (0.3)%, which is 3.1 times higher than the result of **Ene0-C4** but 3.2 times lower than the ε_f_ value of the **Ene1-C4** composite.

Comparing the three families of thermosets and composites, it appeared that the presence of the additional ester linkages in **alkene 1** and **alkyne 1** did not increase the modulus and strength of these materials through secondary H-bonding as expected but that instead they reduced these properties relative to the **alkene 0** materials. However, the addition of the ester linkages did result in increased toughness and strain at the maximum stress of the **Ene1-C4** and **Yne1-C4** composites relative to **Ene0-C4**. The loss in modulus and strength upon the addition of ester linkages was also effectively offset by replacing the alkene groups with alkyne functions, resulting in the very tough **alkyne 1** materials.

Further mechanical evaluations were conducted on the **Ene0-T1**, **Ene1-T1**, and **Yne1-T1** thermosets and composites with the maximum HA concentrations (**Ene0-C4**, **Ene1-C4**, and **Yne1-C4**) to determine how their properties were affected by the absorption of water after being immersed in PBS solution (pH = 7.4) for 7 days (Table 2), which is an important consideration for materials intended for in vivo applications. First, the water absorption results (Figure 2g and Supporting Information Figure S13) revealed that **alkene 0** materials **Ene0-T1** and **Ene0-C4** absorbed the least amount of water [1.30 (0.04) and 1.30 (0.04)%, respectively], while the **alkyne 1** materials **Yne1-T1** and **Yne1-C4** absorbed 1.37 (0.05) and 1.61 (0.05)%, respectively, and the **alkene 1** materials **Ene1-T1** and **Ene1-C4** absorbed the most water [1.55 (0.03) and 2.07 (0.02)%]. This can be explained by the fact that the introduction of ester linkage in the chemical structure of the **alkene 1** and **alkyne 1** monomers facilitated water absorption. This increase in water absorption was likely offset in the **alkyne 1** materials due to their highest cross-link density resulting from TYC instead of TEC chemistry.

Regarding their mechanical properties under wet conditions (Table 2), large decreases in flexural modulus and strength were observed for both the **alkene 0** and **alkyne 1** materials. For instance, the flexural modulus of the **Ene0-C4** composite decreased from 6480 (280) MPa under dry conditions to 3093 (199) MPa after absorbing water, and its flexural strength decreased from 68 (5) to 27 (1) MPa. Meanwhile, the flexural modulus of **Yne1-C4** was reduced by 1.5 times, and it descended from 3480 (231) to 2288 (98) MPa after water absorption. However, for **Ene1-C4**, the flexural modulus and strength remained at the same level, with values of 57 (1) and 4.4 (0.06) MPa, respectively, even though it absorbed more water than the **alkene 0** and **alkyne 1** materials (Figure 2g).

To better understand the vast difference in flexural modulus between the **alkene 0**, **alkene 1**, and **alkyne 1** materials and to study the distribution of HA fillers in the composites, SEM and EDS have been used to image the cross sections for the materials **Ene0-T1**, **Ene0-C4**, **Ene1-T1**, **Ene1-C4**, **Yne1-T1**, and **Yne1-C4**. As can be seen from the SEM images (Figure 2c–e), the composites contain air bubbles which were not observed in the thermosets, which was likely due to the higher viscosity of the composites. To some extent, the presence of bubbles in the composites results in negative impacts regarding the mechanical properties of the materials, and their effect should be further studied in the future. The EDS images show that sulfur is well spread through the cross sections for both composites and thermosets, which indicates a homogeneous distribution of the cross-linking network (Supporting Information, Figure S10). Moreover, from the calcium mapping, it is clear that the HA particles in the composites were also evenly distributed, suggesting that the filler is well integrated throughout the cross-linked networks. Therefore, it appeared that the significant mechanical differences between the materials made from **alkene 0**, **alkene 1**, and **alkyne 1** came from their distinguishing chemical structures.

### 3.3 In Vitro Biocompatibility of the TEC and TYC Materials

**Figure 4:**
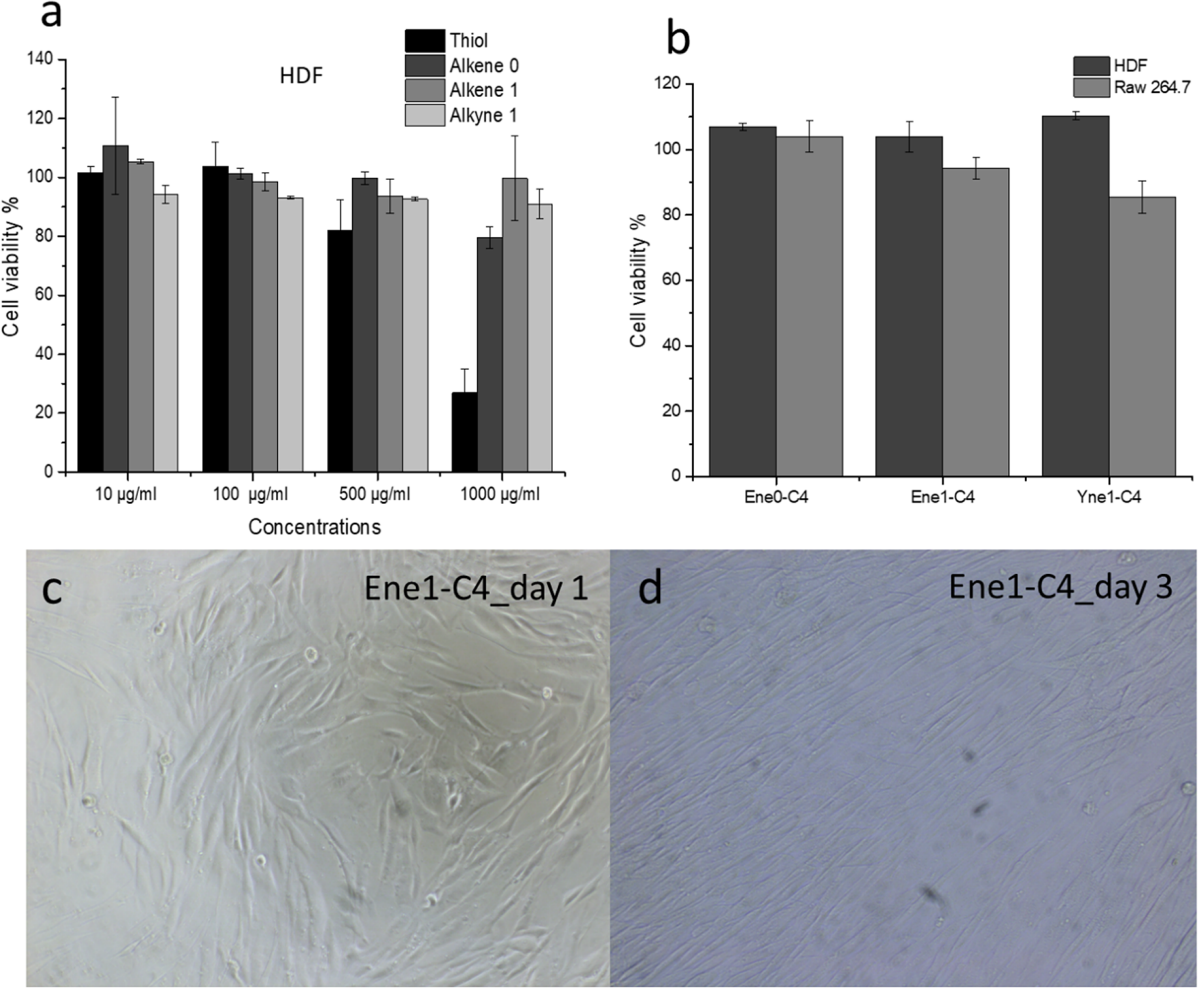
(a) In vitro cytotoxicity evaluation results for monomers **thiol**, **alkene 0**, **alkene 1**, and **alkyne 1** on human dermal fibroblast (HDF) cell culture. The results on Raw 264.7 are given in Supporting Information, Figure S14b; (b) In vitro cytotoxicity evaluation results for the composites **Ene0-C4**, **Ene1-C4**, and **Yne1-C4** (for each with surface area of 3 cm^2^) on both HDF and Raw 264.7 cells. (c,d) Cell viability for materials **Ene1-C4** in HDF on day 1 and day 3.

The biocompatibility of the novel TATO monomers and composites has been tested in vitro. **Alkene 1** and **alkyne 1** showed good biocompatibility toward both human dermal fibroblast (HDF) (Figure 4a, cell viability above 91%) and mouse monocyte cells (Raw 264.7) (Supporting Information, Figure S14b, cell viability above 92%) even at the highest concentration of 1000 μg/mL. **Thiol** showed no obvious cytotoxicity toward HDF (Figure 4a, cell viability above 82%) and Raw 264.7 (Supporting Information, Figure S14b, cell viability above 96%) at concentrations between 10 and 500 μg/mL but was toxic at the high concentration of 1000 μg/mL. **Alkene 0** exhibited good biocompatibility toward HDF, with cell viability above 80% (Figure 4a), and it showed no obvious cytotoxicity toward Raw 264.7 at concentrations between 10 and 500 μg/mL (Supporting Information, Figure S14b). Composites **Ene0-C4**, **Ene1-C4**, and **Yne1-C4** showed excellent biocompatibility toward both HDF and Raw 264.7 (Figure 4b), with cell viability above 104% (**Ene0-C4**), 94% (**Ene1-C4**), and 86% (**Yne1-C4**), respectively. In addition, both HDF (Figure 4c,d and Supporting Information, Figure S15) and Raw 264.7 (Supporting Information, Figure S16) proliferated after 3 days of incubation, indicating that the composites were well-cured, and no toxic components were leached out from the composites.

### 3.4 Fabrication of Objects with the Flexible HA Composites

**Figure 5:**
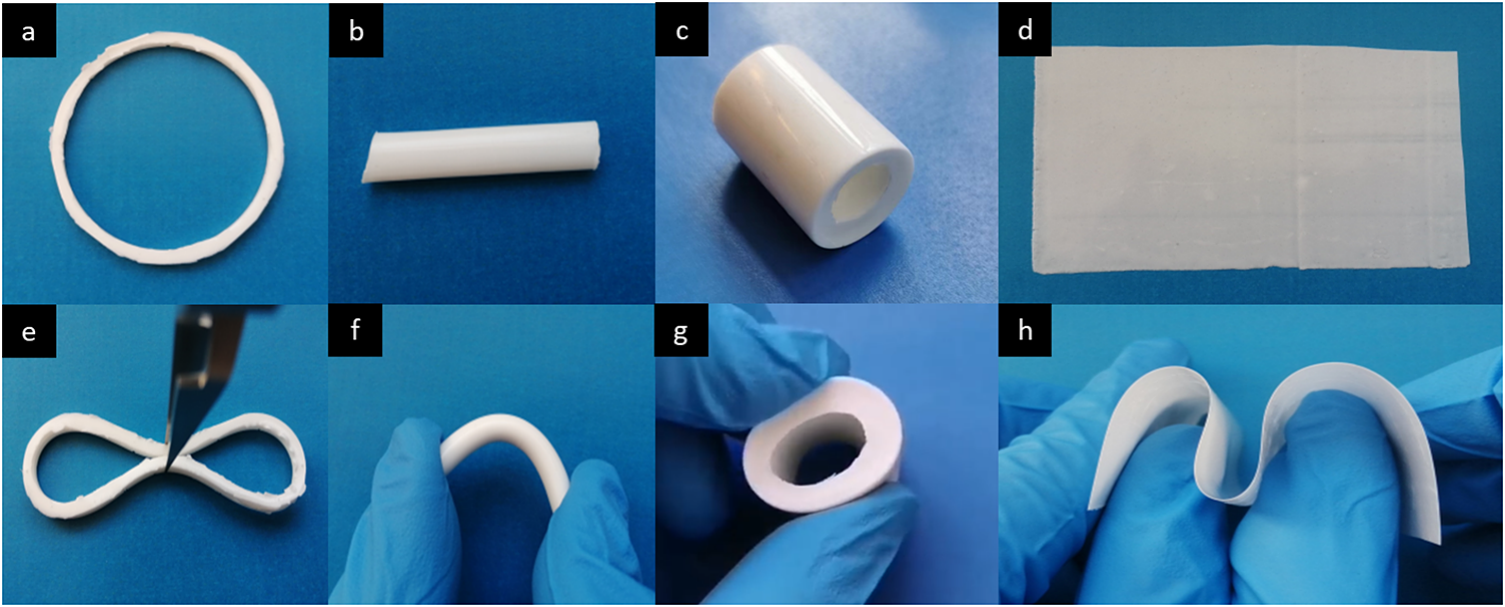
Different objects made by the promising soft composites **Ene1-C4**: (a) ring with a circumference of 126 mm, (b) column with a diameter of 6 mm, (c) tube with a length of 30 mm and wall thickness of 4 mm, and (d) thin film with thickness of 200 μm. The softening properties and the customizable ability are shown in (e–h).

Satisfied with the mechanical and biological properties of these novel materials, we further investigated the development of flexible objects. As a proof of concept, different shapes were produced through drop-casting the composite **Ene1-C4**, which had shown the softest properties of all the composites tested (Figure 5). This proves that, with the composite **Ene1-C4** and different shapes of molds, it is possible to customize and fabricate soft devices of varying shapes, all of which showed an elastic behavior when squeezed or bent with finger pressure (Supporting Information, Objects S1, S2, and S3).

## 4 Conclusions

Four novel trifunctional TATO monomers were synthesized, with the arms containing either ester or amide linkages and terminated with either alkene or alkyne functionalities for use in making photocurable cross-linked networks with thiol-containing monomers through TEC or TYC click chemistry. Formulations were made with the ester-containing monomers and the commercially available **thiol** monomer and varying the concentrations of the HA filler. Unfortunately, the amide-containing monomers proved too insoluble in the thiol and so were not used to make thermosets and composites. It was hypothesized that materials made with these monomers would have enhanced strength due to secondary H-bonding from the esters and that the alkyne monomers would further enhance their modulus due to the increased cross-link density afforded through TYC chemistry. Instead, we discovered that the introduction of the ester linkages resulted in materials that were significantly softer than those made with the previously described **alkene 0** monomer. The HA composites with the ester-containing **alkene 1** monomer were 117 and 10 times softer in terms of flexural modulus and strain at maximum stress than those containing the ester-free **alkene 0** monomer. Comparing the **alkene 1** and **alkyne 1** materials showed that replacing the alkene groups for alkynes resulted in a large increase in modulus and strength, along with a modest increase in cross-link density, as expected. These **alkyne 1** materials displayed the highest toughness but were not as soft as those of **alkene 1**. Mechanical testing under wet conditions indicates that the **alkene 1** composites were able to maintain their mechanical properties after being immersed in PBS (pH = 7.4) for 7 days, while the **alkyne 1** and **alkene 0** composites underwent a significant flexural modulus and strength loss. For all produced composites, in vitro cellular evaluation was conducted, detailing excellent cytotoxicity results. The unique mechanical properties of the **alkene 1** and HA composites were further explored by the fabrication of different proof-of-concept customizable objects. Finally, the combined mechanical, cytotoxicity, and customizability features of **alkene 1-**based cross-linked frameworks have the overarching attributes needed for future biomedical devices in which lower modulus is a prerequisite.

## Supporting Information Available

Demonstration of a thin film. Object S1 (MP4)
Demonstration of a ring. Object S2 (MP4)
Demonstration of a tube. Object S3 (MP4)
Synthesis of monomers, preparation of composite and thermoset materials, characterizations, object demonstrations, and other additional data (PDF)

## Notes

Research data are not shared.
