# Supplementary material for: Soft Hydroxyapatite Composites Based on Triazine–Trione Systems as Potential Biomedical Engineering Frameworks
Source: ACS Appl Mater Interfaces. 2023 Jan 25;15(5):7329–39. doi: 10.1021/acsami.2c16235 (PMC9923673; doi:10.1021/acsami.2c16235)
Supplement: Supplementary file 4 — am2c16235_si_004.pdf [file am2c16235_si_004.pdf]

Supporting information

Soft hydroxyapatite composites based on triazine-trione systems as potential biomedical engineering frameworks

*Jinjian Lin[a], Yanmiao Fan[a], Daniel J. Hutchinson[a], and Michael Malkoch[a]**

[a]J. Lin, Y. Fan, D. J. Hutchinson, Prof. M. Malkoch
KTH Royal Institute of Technology, School of Engineering Sciences in Chemistry, Biotechnology and Health (CBH), Fiber and Polymer Technology, Teknikringen 56-58, SE-100 44 Stockholm, Sweden
E-mail: malkoch@kth.se

1. Synthesis of monomers

*1.1 Synthesis of TESTATO-4PA (**alkene 1**)*

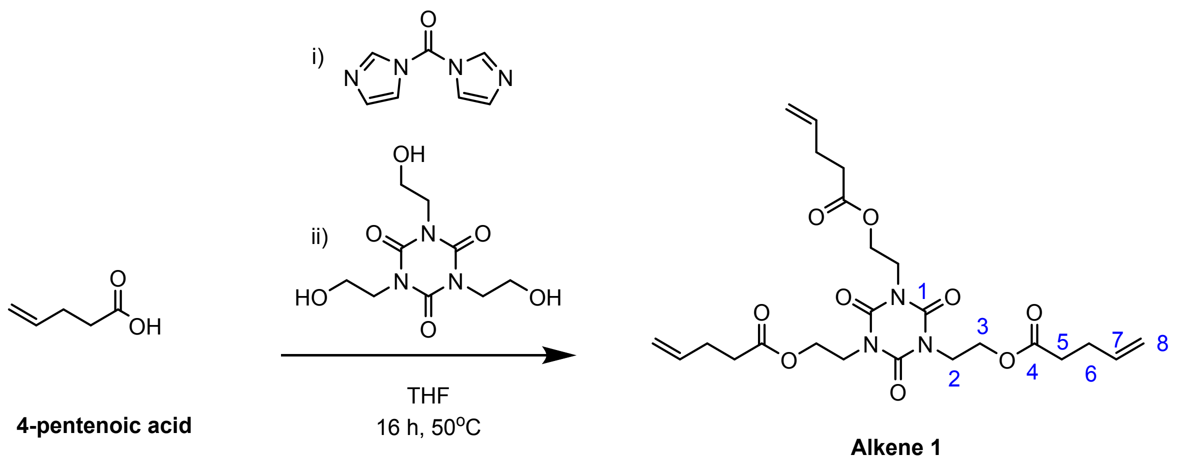

Figure S1. Synthesis of **alkene 1** from tris(2-hydroxyethyl) isocyanurate and 4-pentenoic acid (showing NMR numbering).

Alkene 1: 4-Pentenoic acid (30.2 g, 0.30 mol) was slowly added to THF (400 mL) and the mixture was stirred at 50°C. CDI (51.3 g, 0.32 mol) was then added slowly and the reaction was allowed to react for 2 h. The reaction was monitored by **MALDI**, **^{13}C NMR,** and **^{1}H NMR**. After completion, tris(2-hydroxyethyl) isocyanurate (25.0 g, 0.096 mol) and CsF (8.72 g, 0.057 mol) was added, and the reaction was stirred at 50°C overnight. Afterwards, THF was removed and EtOAc (250 mL) was added. The reaction was then quenched by adding water (200 mL) and cooled to room temperature. The mixture was then washed three times with $NaHCO_3$ 10% solution and $NaHSO_4$ 10% solution. The organic phase was dried over $MgSO_4$, evaporated and dried *in vacuo* to give **alkene 1** as a yellow viscous liquid (44.6 g, 92%). **^{1}H NMR** ($CDCl_3$, 400 MHz), δ/ppm: 5.80 (3H, ddt, *J*=16, 10, 6 Hz, H7), 5.01 (6H, m, H8), 4.32 (6H, dd, *J*=6, 5 Hz, H3), 4.17 (6H, dd, *J*=6, 5 Hz, H2), 2.36 (12H, m, H5 & H6) **^{13}C NMR** ($CDCl_3$, 101 MHz), δ/ppm: 173.01 (C4), 149.06 (C1), 136.69 (C7), 115.65 (C8), 61.11 (C3), 42.19 (C2), 33.38 (C5), 28.71 (C6). **MS (MALDI-TOF)** calculated for $C_{24}H_{33}N_3O_9Na$ [M+Na] 530.54, found 532.38.

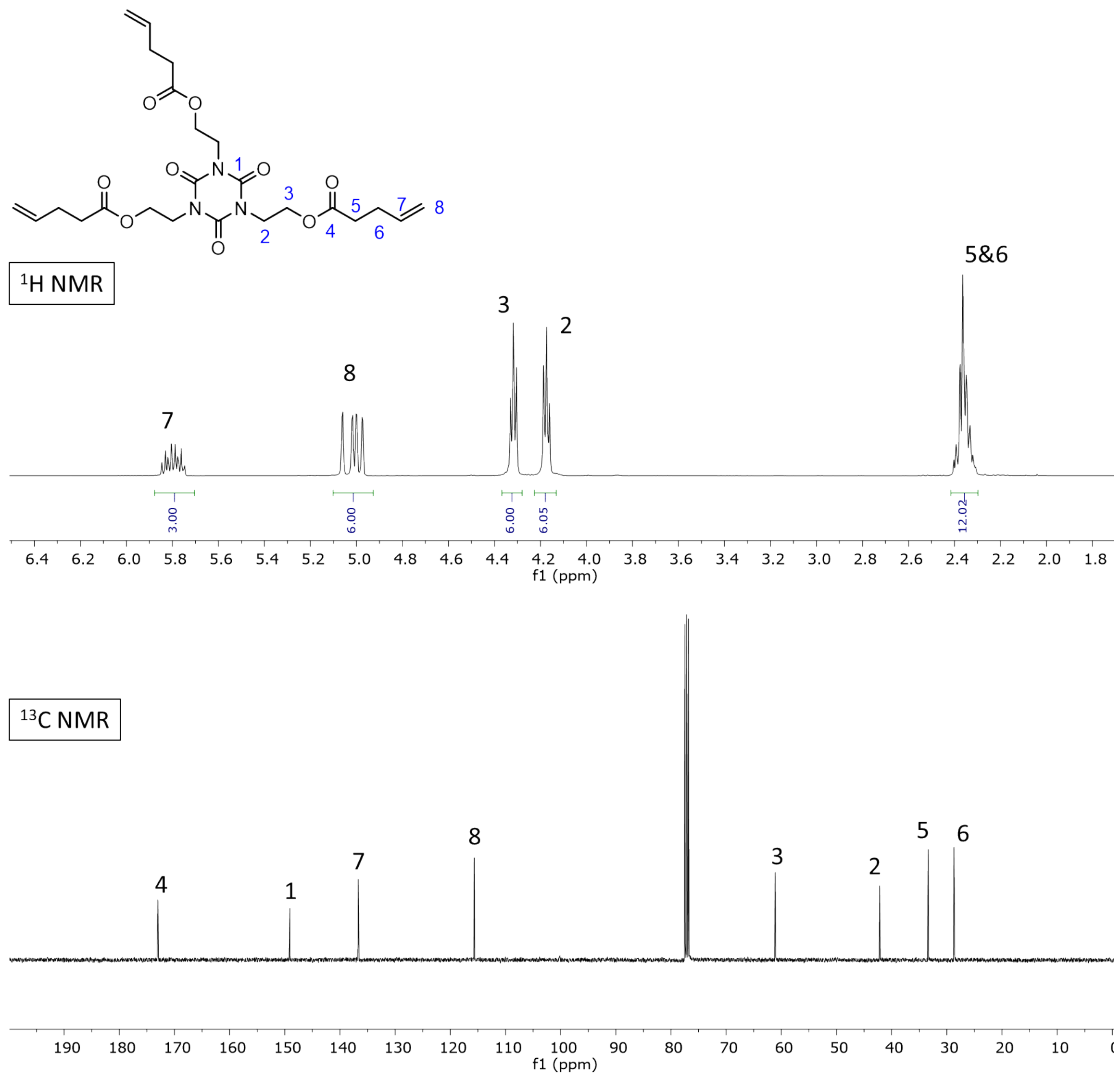

Figure S2. ^{1}H NMR and ^{13}C NMR analysis of monomer **alkene 1** in $CDCl_3$.

1.2 Synthesis of TESTATO-4PTYA ***(alkyne 1)***

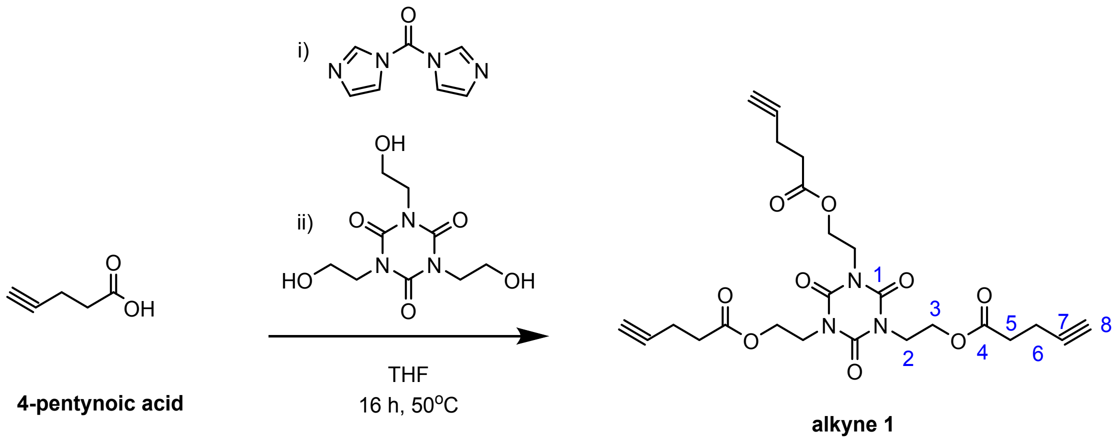

Figure S3. Synthesis of **alkyne 1** from tris(2-hydroxyethyl) isocyanurate and 4-pentynoic acid (showing NMR numbering).

Alkyne 1: 4-Pentynoic acid (9.65 g, 0.10 mol) was slowly added to THF (150 mL), and the mixture was stirred at 50°C. CDI (16.7 g, 0.10 mol) was then added slowly and the reaction was allowed to react for 2 h. The reaction was monitored by **^{13}C NMR** and **^{1}H NMR**. After completion, tris(2-hydroxyethyl) isocyanurate (8.16 g, 0.03 mol) and CsF (2.85 g, 0.02 mol) were added, and the reaction was stirred at 50°C overnight. The reaction was then quenched by adding water (200 mL) and cooled to room temperature. The mixture was then washed three times with $NaHCO_3$ 10% solution and $NaHSO_4$ 10% solution. The organic phase was dried over $MgSO_4$, evaporated and dried *in vacuo* to give **alkyne 1** as yellow viscous liquid (7.80 g, 50%). **^{1}H NMR** (DMSO-D6, 400 MHz), δ/ppm: 4.22 (6H, t, *J*=5 Hz, H3), 4.02 (6H, t, J= 5 Hz, H2), 2.77 (3H, t, *J*=3 Hz, H8), 2.46 (6H, t, *J*=7 Hz, H5), 2.37 (6H, t, J=7 Hz, H6) **^{13}C NMR** (DMSO-D6, 101 MHz), δ/ppm: 171.21 (C4), 148.91 (C1), 82.94 (C7), 71.49 (C8), 60.80 (C3), 41.14 (C2), 32.69 (C5), 13.61 (C6). **MS (MALDI-TOF)** calculated for $C_{24}H_{27}N_3O_9Na$ [M+Na] 524.49, found 525.72.

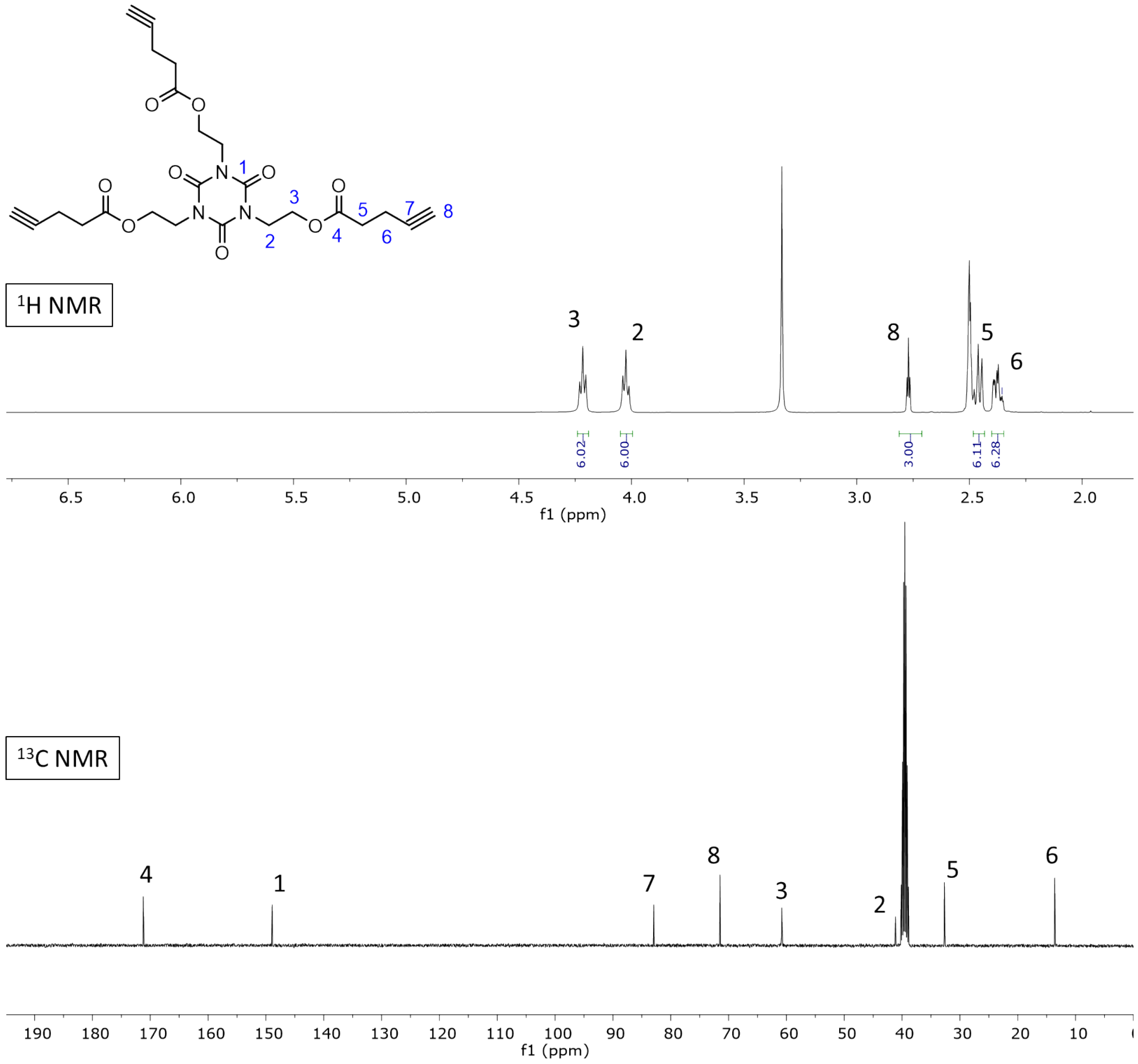

Figure S4. ^{1}H NMR and ^{13}C NMR analysis of monomer **alkyne 1** in DMSO-d6.

*1.3 Synthesis of Tri-amide-TATO core (**TAMTATO**)*

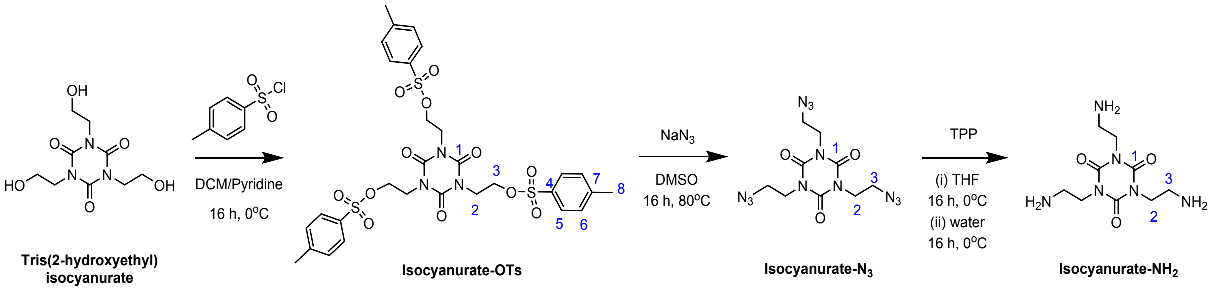

Figure S5. Synthesis of the TATO core (**isocyanurate-NH$_2$**) from tris(2-hydroxyethyl) isocyanurate (showing NMR numbering).

Isocyanurate-OTs: A solution of 4-toluenesulfonyl chloride (263 g, 1.38 mol) in DCM (125 mL) and pyridine (250 mL) was added to a suspension of tris(2-hydroxyethyl) isocyanurate (100 g, 0.383 mol) in DCM (125 mL). The mixture was cooled to 0°C then allowed to react

overnight with stirring. Ice (200 g) was then added to the solution, and the product was extracted with DCM. The organic extracts were combined, dried over $MgSO_4$, evaporated and dried *in vacuo* to give **isocyanurate-OTs** as a white solid (261 g, 94%). **^{1}H NMR** ($CDCl_3$, 400 MHz), δ/ppm: 7.75 (6H, m, H5), 7.32 (6H, d, *J*=8 Hz, H6), 4.26 (6H, t, *J*=5Hz, H3), 4.13 (6H, t, *J*=5 Hz, H2), 2.43 (9H, s, H8). **^{13}C NMR** ($CDCl_3$, 101 MHz), δ/ppm: 148.4 (C1), 145.2 (C4), 132.7 (C7), 130.0 (C6), 127.9 (C5), 65.9 (C3), 41.6 (C2), 21.7 (C8).

Isocyanurate-N_3: Isocyanurate-OTs (55.0 g, 0.076 mol) was dissolved in DMSO (350 mL). NaN_3 (23.2 g, 0.357 mol) was then added and the solution was stirred at 80°C overnight. The reaction mixture was then cooled to room temperature and $NaHCO_3$ 10% solution (150 mL) was added. The mixture was cooled in an ice bath for 10 minutes, then EtOAc (200 mL) was added. The solution was then extracted with further EtOAc. The EtOAc fractions were combined and washed with water to remove DMSO. They were then dried over $MgSO_4$, evaporated and dried *in vacuo* to give **Isocyanurate-N_3** as a white solid (21.0 g, 82%). **^{1}H NMR** ($CDCl_3$, 400 MHz), δ/ppm: 4.16 (6H, t, *J*=6 Hz, H2), 3.58 (6H, t, *J*=6 Hz, H3). **^{13}C NMR** ($CDCl_3$, 101 MHz), δ/ppm: 148.8 (C1), 48.5 (C2/3).

Isocyanurate-NH_2: A solution of isocyanurate-N_3 (21.0 g, 0.062 mol) in THF (250 mL) was cooled to 0°C in an ice bath. Triphenylphosphine (TPP, 81.9 g, 0.312 mol) was slowly added at 0°C. The reaction mixture was then stirred overnight at room temperature. Water (100 mL) was added, the reaction was cooled to 0°C and then allowed to reaction overnight at room temperature again. THF was then removed by evaporation and more water (300 mL) was added. The aqueous fraction was then washed with toluene (4 x 300 mL) before being freeze dried to give **isocyanurate-NH_2** as a white solid (13.2 g, 82%). **^{1}H NMR** (DMSO-d6, 400 MHz), δ/ppm: 3.74 (6H, t, *J*=7 Hz, H2), 2.69 (6H, t, J=7 Hz, H3). **^{13}C NMR** (MeOD, 101 MHz), δ/ppm: 151.5 (C1), 45.8 (C2), 40.5 (C3).

*1.4 Synthesis of TAMTATO-4PA (**alkene 2**)*

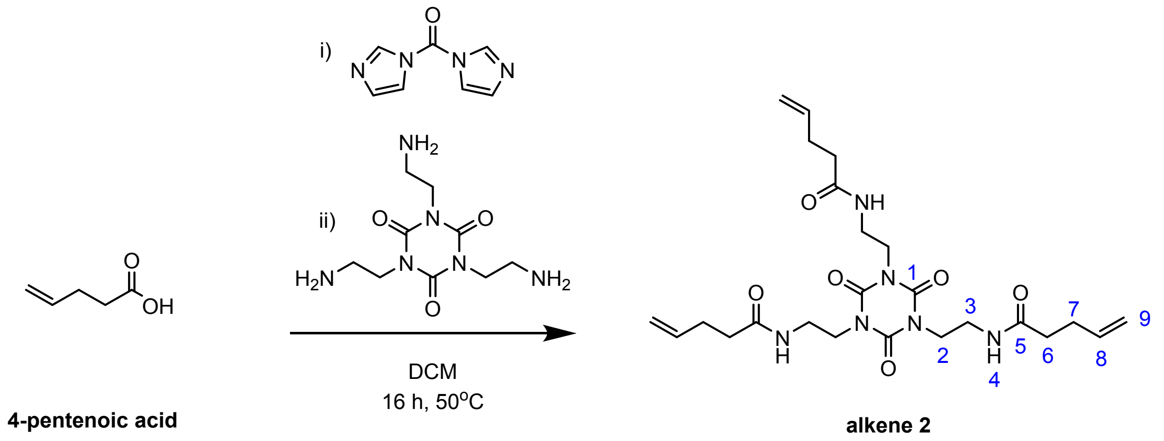

Figure S6. Synthesis of **alkene 2** from isocyanurate-NH_2 and 4-pentenoic acid (showing NMR numbering).

Alkene 2): 4-Pentenoic acid (1.83 g, 0.018 mol) was slowly added to DCM (60 mL) and the mixture was stirred at 50°C. CDI (3.26 g, 0.02 mol) was then added slowly and the reaction was allowed to react for 2 h. The reaction was monitored by **^{13}C NMR** and **^{1}H NMR**. After completion, isocyanurate-NH_2 (1.50 g, 0.006 mol) and CsF (0.53 g, 0.003 mol) was added and the reaction was stirred at 50°C overnight. The reaction was then quenched by adding water (50 mL) and cooled to room temperature. The mixture was then washed three times with $NaHCO_3$ 10% solution and $NaHSO_4$ 10% solution. The organic phase was dried over $MgSO_4$, evaporated and dried *in vacuo* to give **alkene 2** as a dark yellow solid (1.27 g, 42%). **^{1}H NMR** (DMSO-d6, 400 MHz), δ/ppm: 7.93 (3H, t, *J*=6 Hz, H4), 5.76 (3H, ddt, *J*=6, 10, 17 Hz, H8), 4.99 (3H, dd, *J*=2, 17 Hz, H9a), 4.92 (3H, dd, *J*=2, 10 Hz, H9b), 3.82 (7H, t, *J*=6 Hz, H3), 3.28 (9H, q, *J*=6 Hz, H2), 2.19 (9H, q, *J*=7 Hz, H6), 2.08 (7H, q, *J*=8 Hz, H7). **^{13}C NMR** (DMSO-d6, 101 MHz), δ/ppm: 171.9 (C5), 149.0 (C1), 137.6 (C8), 114.9 (C9), 41.8 (C2), 35.9 (C6), 34.6 (C3), 29.1 (C7). **MS (MALDI-TOF)** calculated for $C_{24}H_{36}N_6O_6Na$ [M+Na] 527.59, found 528.15.

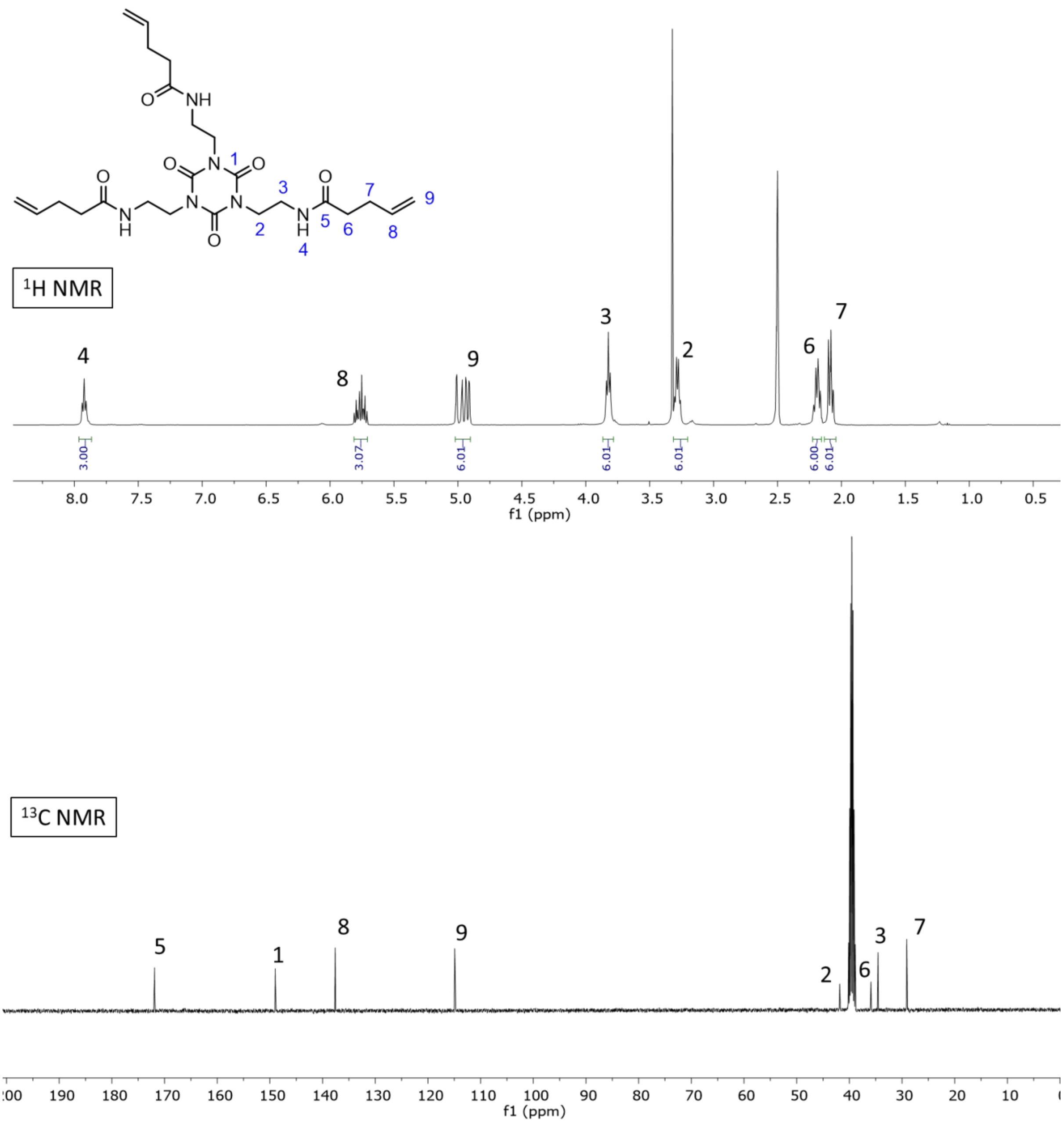

Figure S7. ^{1}H NMR and ^{13}C NMR analysis of monomer **alkene 2** in DMSO-d6.

*1.5 Synthesis of TAMTATO-4PTYA (**alkyne 2**)*

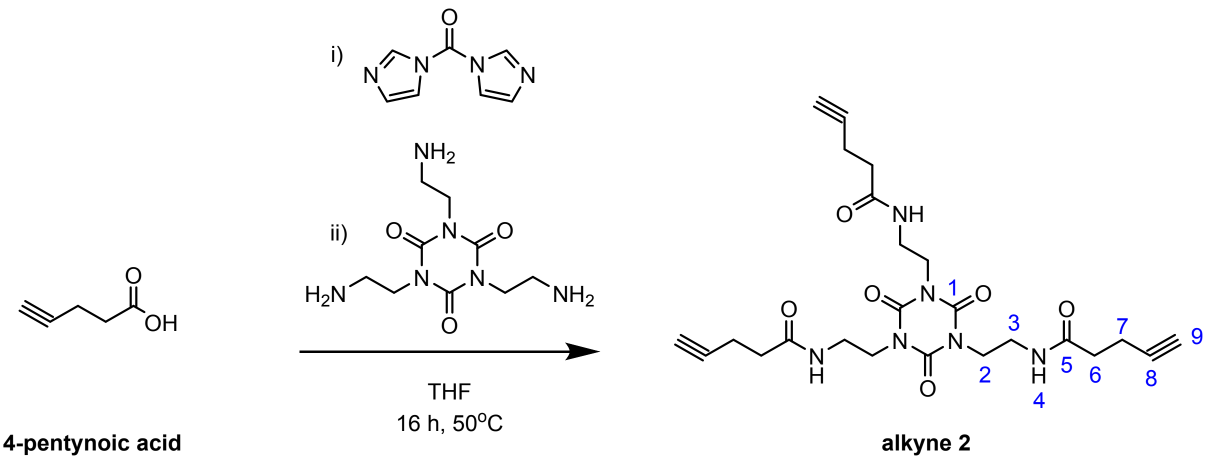

Figure S8. Synthesis of **alkyne 2** from isocyanurate-NH_2 and 4-pentynoic acid (showing NMR numbering).

Alkyne 2: 4-Pentynoic acid (4.83 g, 0.049 mol) was slowly added to THF (100 mL) and the mixture was stirred at 50°C. CDI (8.39 g, 0.05 mol) was then added slowly and the reaction was allowed to react for 2 h. The reaction was monitored by **^{13}C NMR** and **^{1}H NMR**. After completion, isocyanurate-NH_2 (4.04 g, 0.0156 mol) and CsF (1.42 g, 0.009 mol) was added and the reaction was stirred at 50°C overnight. The other day, the reaction was cooled to room temperature. THF was evaporated and EtOAc (100mL) was added. The reaction was then quenched by adding water (100 mL). The product came out as solid in the solution and it was filtered off and then washed by water for 3 times. The solid was then collected and freeze dried to remove water and give white solid as **alkyne 2** (3.41 g, 44%). **^{1}H NMR** (DMSO-d6, 400 MHz), δ/ppm: 7.98 (3H, t, *J*=6 Hz, H4), 3.82 (6H, t, *J*=6 Hz, H3), 3.28 (6H, q, *J*=6 Hz, H2), 2.74 (3H, t, *J*=2 Hz, H9), 2.32 (6H, td, *J*=7, 2 Hz, H7), 2.20 (6H, t, *J*=7 Hz, H6). **^{13}C NMR** (DMSO-d6, 101 MHz), δ/ppm: 170.69 (C5), 149.0 (C1), 83.61 (C8), 71.28 (C9), 41.73 (C2), 36.02 (C3), 34.23 (C6), 14.08 (C7). **MS (MALDI-TOF)** calculated for $C_{24}H_{30}N_6O_6Na$ [M+Na] 521.53, found 521.27.

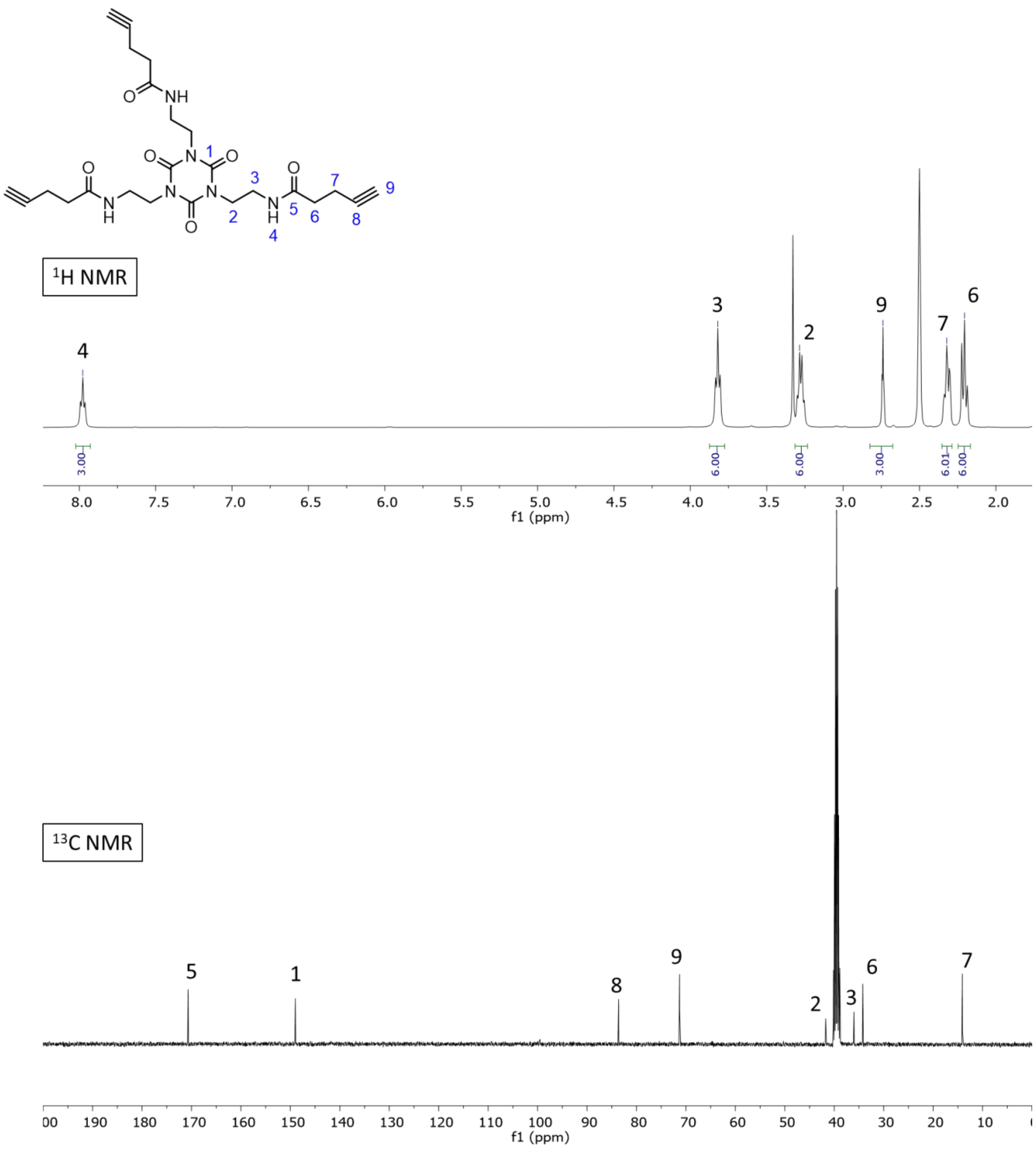

Figure S9. ^{1}H NMR and ^{13}C NMR analysis of monomer **alkyne 2** in DMSO-d6.

2. SEM and EDS images of cross-section

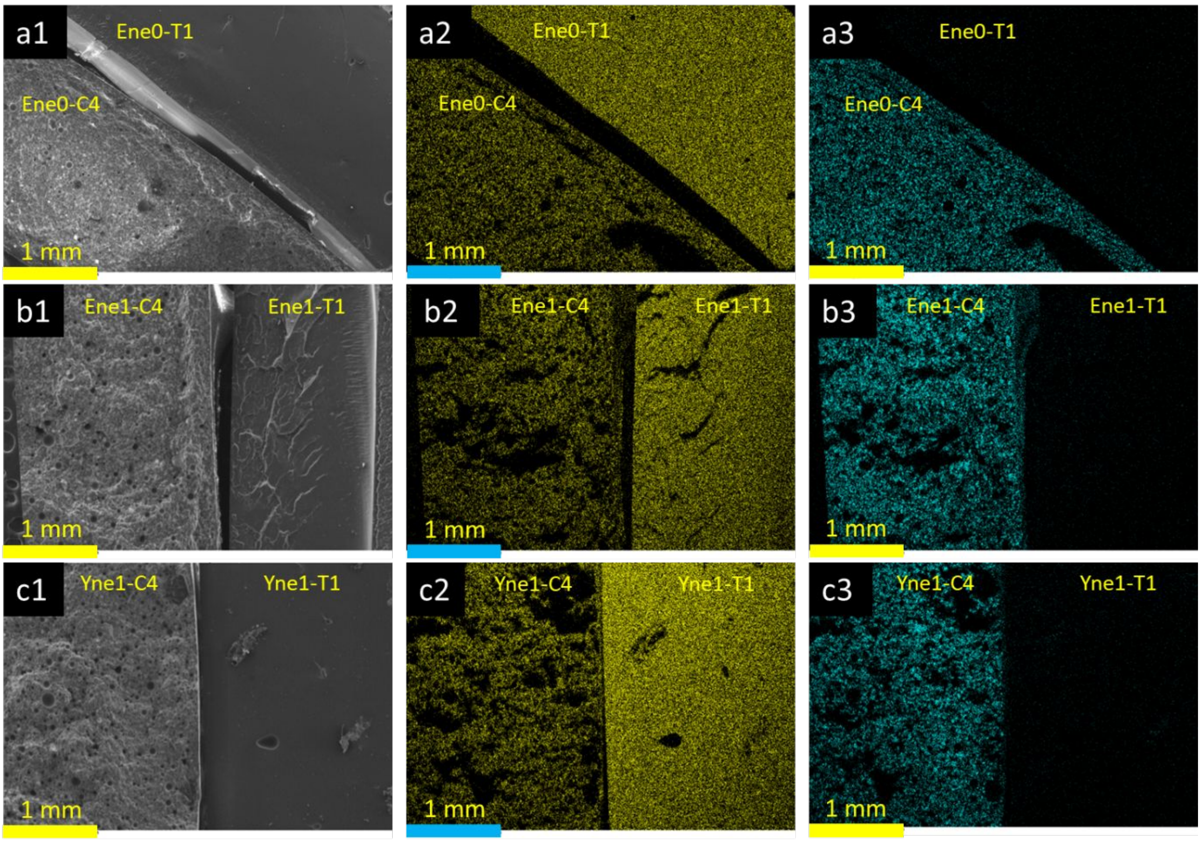

Figure S10. SEM (a1, b1 and c1) and EDS images of **S** (a2, b2 and c2) and **Ca** (a3, b3 and c3) elements for materials **Ene0-T1**, **Ene0-C4**, **Ene1-T1**, **Ene1-C4**, **Yne1-T1**, and **Yne1-C4**, respectively. The scale of measurement is 1 mm.

3. Object demonstrations

Three mp4 videos, which are called **object S1. thin film**, **object S2. ring** and **object S3. tube**, have been recorded to show the softness and the flexibility of the fabricated objects (including a thin film, a ring, and a tube) at which they returned to the original shape after getting squeezed or folded. The thin film is with thickness of 200 µm, the ring is with a circumference of 126 mm and the tube is with length of 30 mm and wall thickness of 4 mm.

4. Preparation of composite and thermoset materials *4.1 Preparation of **Ene0-T1** material*

TEMPIC (2.11 g, 4.01 mmol) and **alkene 0** (1.00g, 4.01 mmol) were added into a glass vial. Aluminum foil was used to cover the vial and then TPO (0.0178 g, 0.05 mmol) was added. The compounds were then mixed by a glass pipette to ensure a homogeneous mixture. The resin

was then transferred to a beam-shaped silicon mold with dimensions of 32 mm x 6 mm x 2 mm and cured by handheld LED lamp Bluephase 20i to give **Ene0-T1**.

4.2 Preparation of ***Ene0-C2*** *material*

TEMPIC (2.11 g, 4.01 mmol) and **alkene 0** (1.00g, 4.01 mmol) were added into a glass vial. Aluminum foil was used to cover the vial and then TPO (0.0178 g, 0.05 mmol) was added. The compounds were then mixed by a glass pipette to ensure homogeneous mixture. HA (0.715 g, 1.42 mmol) was then added, and the compounds were mixed again before transfer to a beam-shaped silicon mold with dimensions of 32 mm x 6 mm x 2 mm. Handheld LED light Bluephase 20i was then used to cure the resin to give **Ene0-C2**.

4.3 Preparation of ***Ene0-C3*** *material*

TEMPIC (2.11 g, 4.01 mmol) and **alkene 0** (1.00g, 4.01 mmol) were added into a glass vial following the order. Aluminum foil was used to cover the vial and then TPO (0.0178 g, 0.05 mmol) was added. The compounds were then mixed by a glass pipette to ensure homogeneous mixture. HA (1.855 g, 3.6929 mmol) was then added, and the compounds were mixed again before transfer to a beam-shape silicon mold with dimensions of 32 mm x 6 mm x 2 mm. Handheld LED light Bluephase 20i was then used to cure the resin to give **Ene0-C3**.

4.4 Preparation of ***Ene0-C4*** *material*

TEMPIC (2.11 g, 4.01 mmol) and **alkene 0** (1.00g, 4.01 mmol) were added into a glass vial following the order. Aluminum foil was used to cover the vial and then TPO (0.0178 g, 0.05 mmol) was added. The compounds were then mixed by a glass pipette to ensure homogeneous mixture. HA (4 g, 7.96 mmol) was then added, and the compounds were mixed again before transfer to a beam-shape silicon mold with dimensions of 32 mm x 6 mm x 2 mm. Handheld LED light Bluephase 20i was then used to cure the resin to give **Ene0-C4**.

4.5 Preparation of ***Ene1-T1*** *material*

TEMPIC (2.11 g, 4.01 mmol) and **alkene 1** (2.037g, 4.01 mmol) were added into a glass vial following the order. Aluminum foil was used to cover the vial and then TPO (0.0178 g, 0.05 mmol) was added. The compounds were then mixed by a glass pipette to ensure homogeneous mixture. The resin was then transferred to a beam-shape silicon mold with dimensions of 32 mm x 6 mm x 2 mm and cured by handheld LED lamp Bluephase 20i to give **Ene1-T1**.

*4.6 Preparation of **Ene1-C2** material*

TEMPIC (2.11 g, 4.01 mmol) and **alkene 1** (2.037g, 4.01 mmol) were added into a glass vial following the order. Aluminum foil was used to cover the vial and then TPO (0.0178 g, 0.05 mmol) was added. The compounds were then mixed by a glass pipette to ensure homogeneous mixture. HA (1.045 g, 2.0804 mmol) was then added, and the compounds were mixed again before transfer to a beam-shape silicon mold with dimensions of 32 mm x 6 mm x 2 mm. Handheld LED light Bluephase 20i was then used to cure the resin to give **Ene1-C2**.

*4.7 Preparation of **Ene1-C3** material*

TEMPIC (2.11 g, 4.01 mmol) and **alkene 1** (2.037g, 4.01 mmol) were added into a glass vial following the order. Aluminum foil was used to cover the vial and then TPO (0.0178 g, 0.05 mmol) was added. The compounds were then mixed by a glass pipette to ensure homogeneous mixture. HA (2.8 g, 5.5742 mmol) was then added, and the compounds were mixed again before transfer to a beam-shape silicon mold with dimensions of 32 mm x 6 mm x 2 mm. Handheld LED light Bluephase 20i was then used to cure the resin to give **Ene1-C3**.

*4.8 Preparation of **Ene1-C4** material*

TEMPIC (2.11 g, 4.01 mmol) and **alkene 1** (2.037g, 4.01 mmol) were added into a glass vial following the order. Aluminum foil was used to cover the vial and then TPO (0.0178 g, 0.05 mmol) was added. The compounds were then mixed by a glass pipette to ensure homogeneous mixture. HA (6.25 g, 12.44 mmol) was then added, and the compounds were mixed again before transfer to a beam-shape silicon mold with dimensions of 32 mm x 6 mm x 2 mm. Handheld LED light Bluephase 20i was then used to cure the resin to give **Ene1-C4**.

*4.9 Preparation of **Yne1-T1** material*

Alkyne 1 (0.86g, 1.72 mmol) and **thiol** (1.810 g, 3.44 mmol) were added into a glass vial following the order. Aluminum foil was used to cover the vial and then TPO (0.0534 g, 0.15 mmol) was added. The compounds were then mixed by a glass pipette to ensure homogeneous mixture. The resin was then transferred to a beam-shape silicon mold with dimensions of 32 mm x 6 mm x 2 mm and cured by handheld LED lamp Bluephase 20i to give **Yne1-T1**.

4.10 Preparation of ***Yne1-C2*** *material*

Alkyne 1 (0.86g, 1.72 mmol) and **thiol** (1.810 g, 3.44 mmol) were added into a glass vial following the order. Aluminum foil was used to cover the vial and then TPO (0.0534 g, 0.15 mmol) was added. The compounds were then mixed by a glass pipette to ensure homogeneous mixture. HA (0.653 g, 1.300 mmol) was then added, and the compounds were mixed again before transfer to a beam-shape silicon mold with dimensions of 32 mm x 6 mm x 2 mm. Handheld LED light Bluephase 20i was then used to cure the resin to give **Yne1-C2**.

4.11 Preparation of ***Yne1-C3*** *material*

Alkyne 1 (0.86g, 1.72 mmol) and **thiol** (1.810 g, 3.44 mmol) were added into a glass vial following the order. Aluminum foil was used to cover the vial and then TPO (0.0534 g, 0.15 mmol) was added. The compounds were then mixed by a glass pipette to ensure homogeneous mixture. HA (1.718 g, 3.420 mmol) was then added, and the compounds were mixed again before transfer to a beam-shape silicon mold with dimensions of 32 mm x 6 mm x 2 mm. Handheld LED light Bluephase 20i was then used to cure the resin to give **Yne1-C3**.

4.12 Preparation of ***Yne1-C4*** *material*

Alkyne 1 (0.86g, 1.72 mmol) and **thiol** (1.810 g, 3.44 mmol) were added into a glass vial following the order. Aluminum foil was used to cover the vial and then TPO (0.0534 g, 0.15 mmol) was added. The compounds were then mixed by a glass pipette to ensure homogeneous mixture. HA (3.66 g, 7.28 mmol) was then added, and the compounds were mixed again before transfer to a beam-shape silicon mold with dimensions of 32 mm x 6 mm x 2 mm. Handheld LED light Bluephase 20i was then used to cure the resin to give **Yne1-C4**.

5. Raman spectra

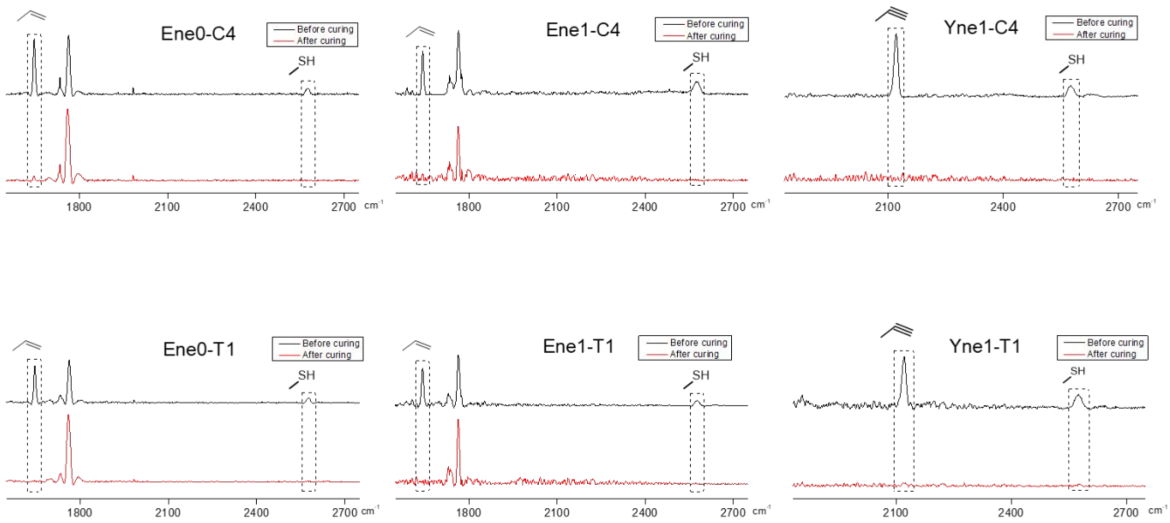

Figure S11. Raman spectra for materials **Ene0-T1**, **Ene0-C4**, **Ene1-T1**, **Ene1-C4**, **Yne1-T1** and **Yne1-C4**, at which the raman shifts at 1630-1660 cm^{-1} (C-C double bond), 2100-2136 cm^{-1} (C-C triple bonds) and 2560-2600 cm^{-1} (-SH) disappeared after the curing and thus, the full TEC or TYC monomers conversion is confirmed.

6. Crosslink density

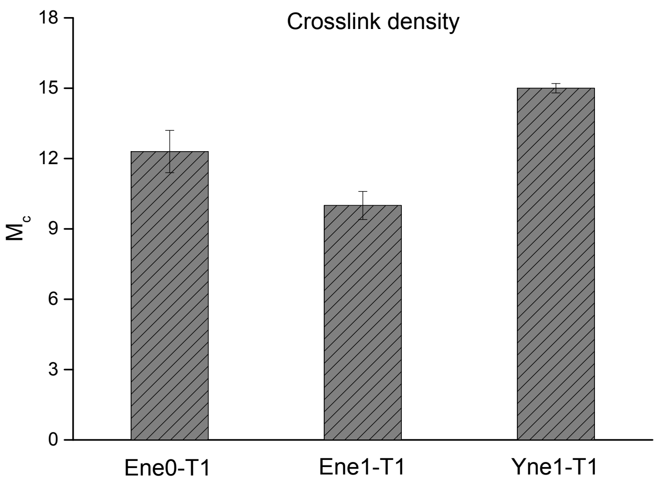

Figure S12. Crosslink density (calculated by DMA results) for thermosets **Ene0-T1**, **Ene1-T1** and **Yne1-T1**.

7. Water absorption and water solubility

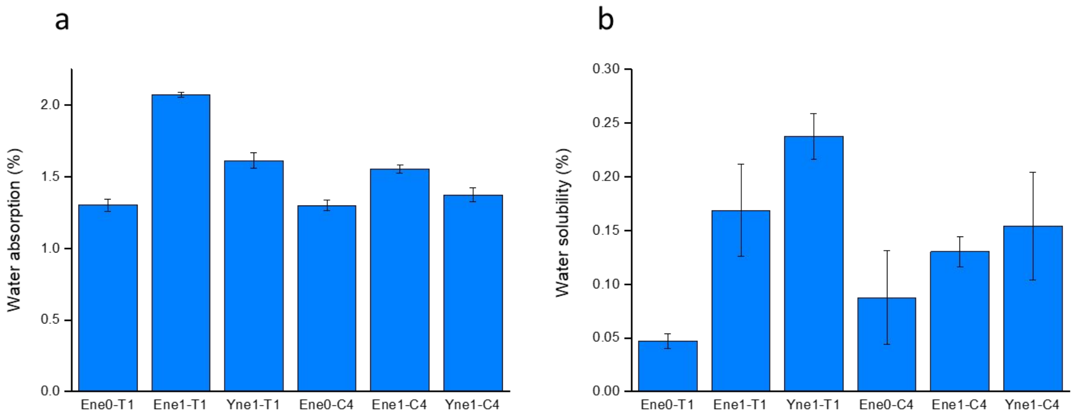

Figure S13. Water absorption (a) and solubility (b) results for materials **Ene0-T1**, **Ene0-C4**, **Ene1-T1**, **Ene1-C4**, **Yne1-T1** and **Yne1-C4** after being immersed in PBS (pH=7.4) solution for 7 days.

8. Cell viability

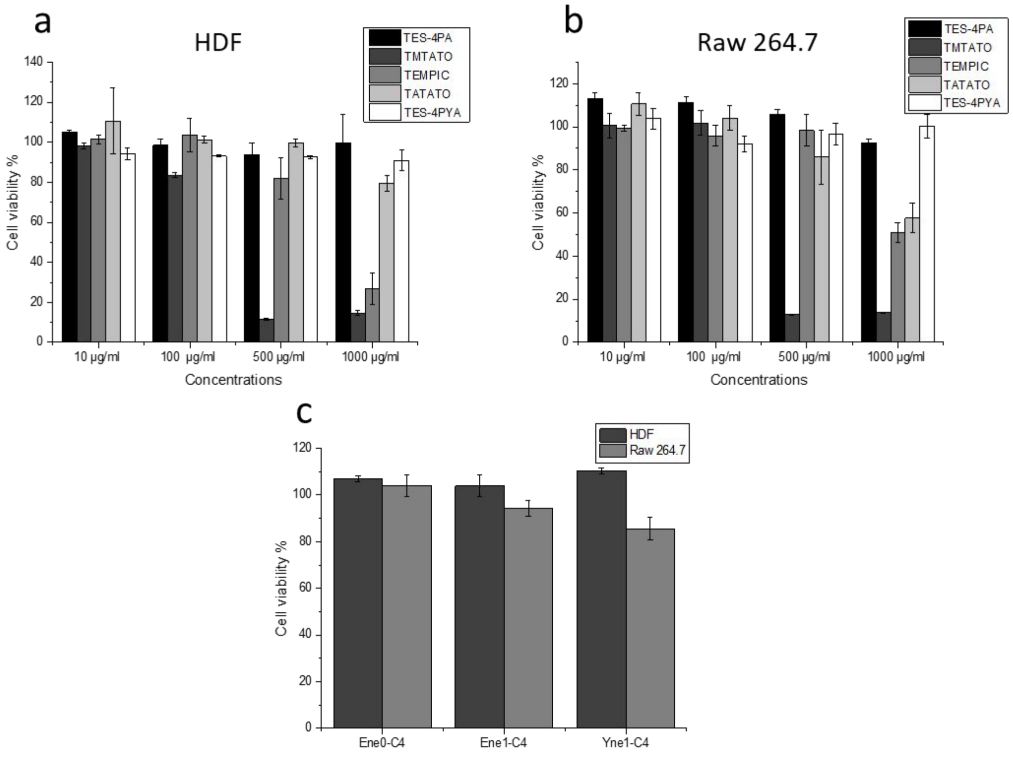

Figure S14. (a) and (b): cell viability for different monomers **thiol**, **alkene 1**, **alkene 2** and **alkyne 1** on two different cell types, HDF and Raw 264.7. (c): cell viability of different materials **Ene0-C4**, **Ene1-C4** and **Yne1-C4** on both HDF and Raw 264.7 cells.

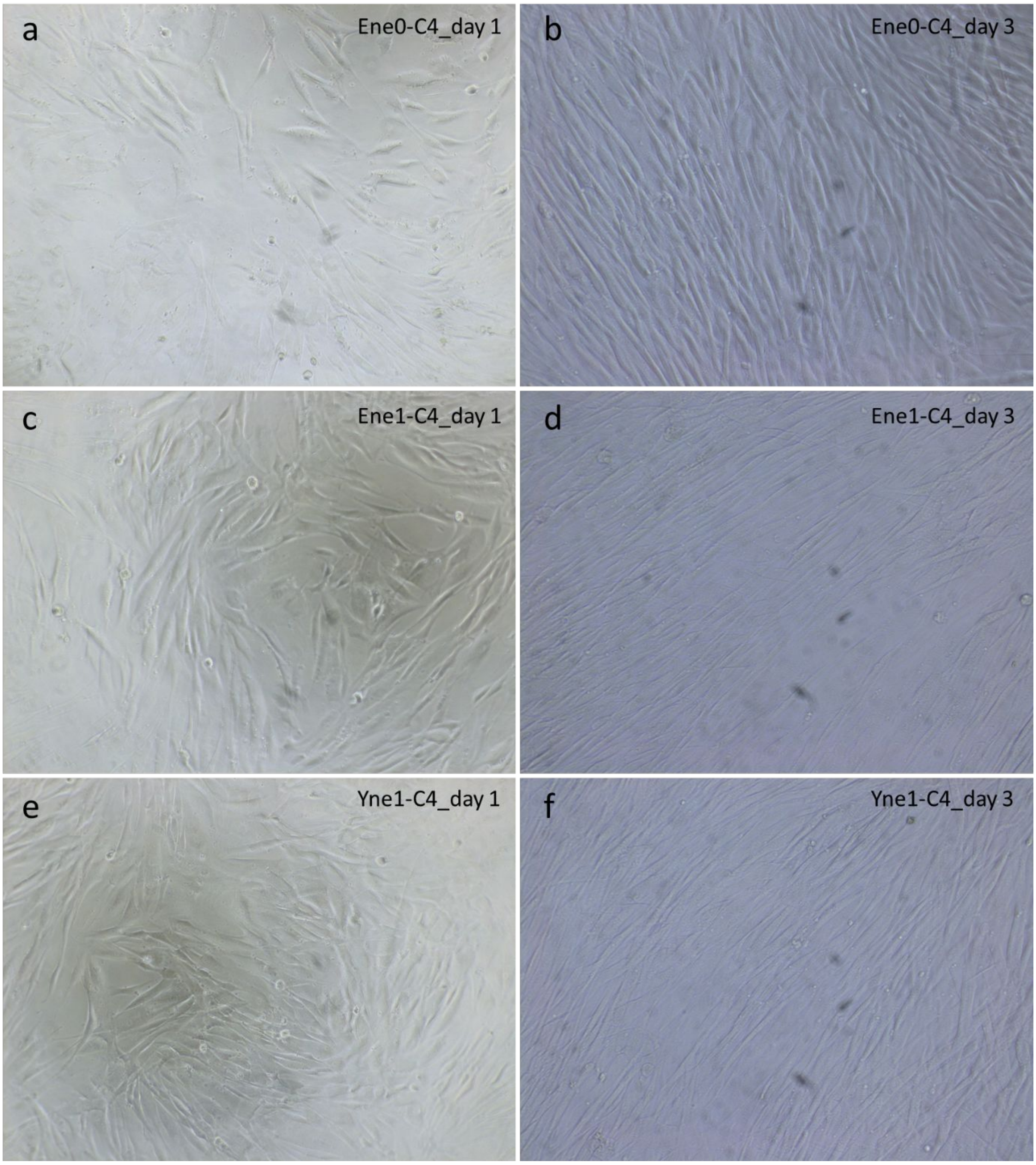

Figure S15. Captured images of cell viability for materials **Ene0-C4**, **Ene1-C4** and **Yne1-C4** in HDF in day 1 and day 3. (a)-(b): cell viability for material **Ene0-C4** in day 1 and day 3. (c)-(d): cell viability for material **Ene1-C4** in day 1 and day 3. (e)-(f): cell viability for material **Yne1-C4** in day 1 and day 3.

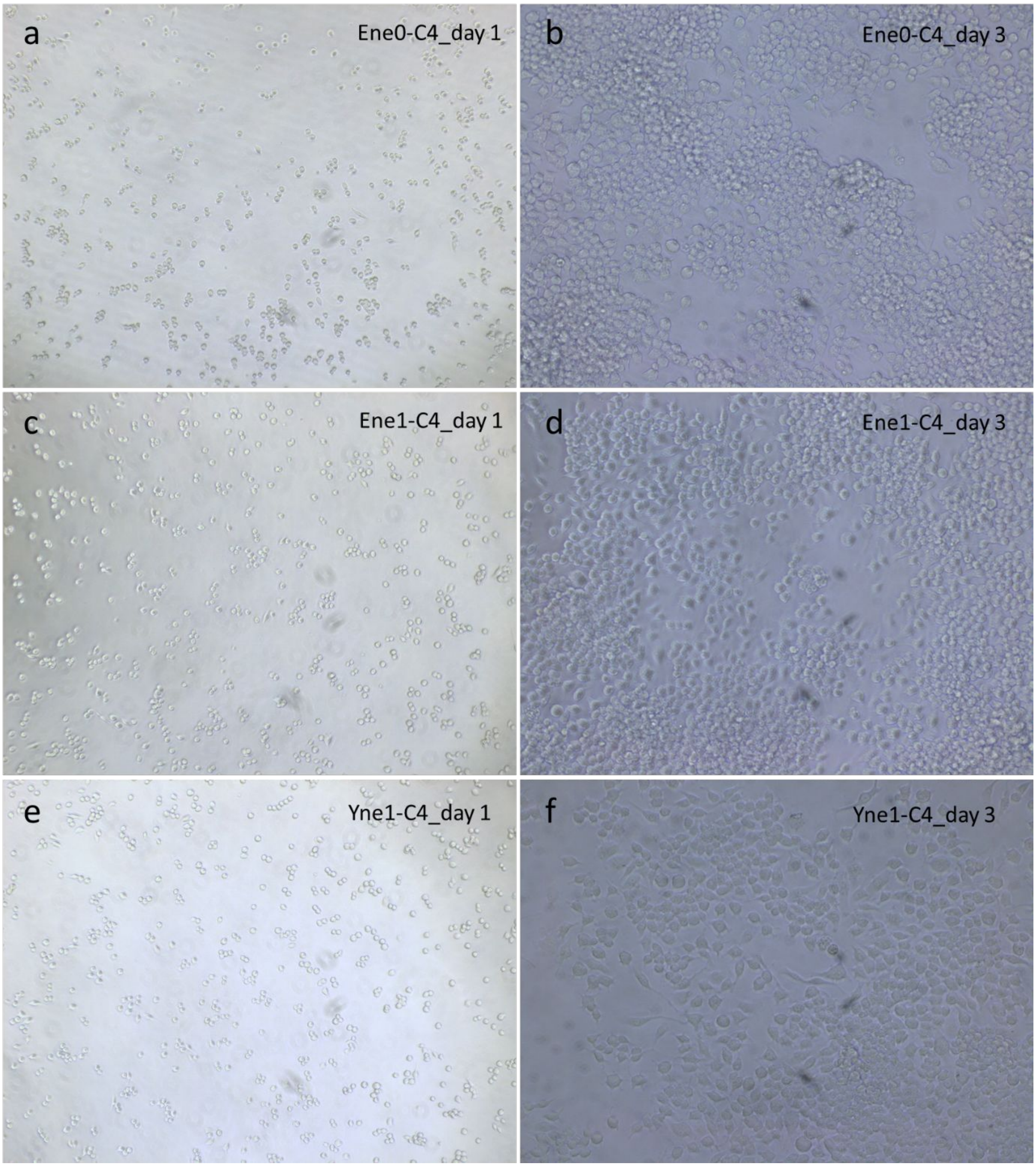

Figure S16. Captured images of cell viability for materials **Ene0-C4**, **Ene1-C4** and **Yne1-C4** in Raw 264.7 in day 1 and day 3. (a)-(b): cell viability for material **Ene0-C4** in day 1 and day 3. (c)-(d): cell viability for material **Ene1-C4** in day 1 and day 3. (e)-(f): cell viability for material **Yne1-C4** in day 1 and day 3.
